# Association between temperature variability and daily hospital admissions for cause-specific cardiovascular disease in urban China: A national time-series study

**DOI:** 10.1371/journal.pmed.1002738

**Published:** 2019-01-28

**Authors:** Yaohua Tian, Hui Liu, Yaqin Si, Yaying Cao, Jing Song, Man Li, Yao Wu, Xiaowen Wang, Xiao Xiang, Juan Juan, Libo Chen, Chen Wei, Pei Gao, Yonghua Hu

**Affiliations:** 1 Department of Epidemiology and Biostatistics, School of Public Health, Peking University, Beijing, China; 2 Medical Informatics Center, Peking University, Beijing, China; 3 Beijing HealthCom Data Technology Co. Ltd, Beijing, China; 4 Key Laboratory of Molecular Cardiovascular (Peking University), Ministry of Education, Beijing, China; Stanford University, UNITED STATES

## Abstract

**Background:**

Epidemiological studies have provided compelling evidence of associations between ambient temperature and cardiovascular disease. However, evidence of effects of daily temperature variability on cardiovascular disease is scarce and mixed. We aimed to examine short-term associations between temperature variability and hospital admissions for cause-specific cardiovascular disease in urban China.

**Methods and findings:**

We conducted a national time-series analysis in 184 cities in China between 2014 and 2017. Data on daily hospital admissions for ischemic heart disease, heart failure, heart rhythm disturbances, and ischemic stroke were obtained from the database of Urban Employee Basic Medical Insurance (UEBMI) including 0.28 billion enrollees. Temperature data were acquired from the China Meteorological Data Sharing Service Center. Temperature variability was calculated from the standard deviation (SD) of daily minimum and maximum temperatures over exposure days. City-specific associations between temperature variability and cardiovascular disease were examined with overdispersed Poisson models controlling for calendar time, day of the week, public holiday, and daily mean temperature and relative humidity. Random-effects meta-analyses were performed to obtain national and regional average associations. We also plotted exposure-response relationship curve using a natural cubic spline of temperature variability. There were 8.0 million hospital admissions for cardiovascular disease during the study period. At the national-average level, a 1-°C increase in temperature variability at 0–1 days (TV_0–1_) was associated with a 0.44% (0.32%–0.55%), 0.31% (0.20%–0.43%), 0.48% (0.01%–0.96%), 0.34% (0.01%–0.67%), and 0.82% (0.59%–1.05%) increase in hospital admissions for cardiovascular disease, ischemic heart disease, heart failure, heart rhythm disturbances, and ischemic stroke, respectively. The estimates decreased but remained significant when controlling for ambient fine particulate matter (PM_2.5_), NO_2_, and SO_2_ pollution. The main limitation of the present study was the unavailability of data on individual exposure to temperature variability.

**Conclusions:**

Our findings suggested that short-term temperature variability exposure could increase the risk of cardiovascular disease, which may provide new insights into the health effects of climate change.

## Abstract 2

Yonghua Hu and colleagues, from their large time-series, cohort analysis of 184 Chinese cities, show that temperature variability in associated with increased cardiovascular-related hospital admissions

## Author summary

**Why was this study done?:**

- Epidemiological studies have reported associations between daily temperature and mortality or morbidity from cardiovascular disease.
- Evidence of effects of temperature variability on cardiovascular disease is scarce and mixed.

**What did the researchers do and find?:**

- We conducted a national time-series study using data on 8.0 million hospital admissions for cause-specific cardiovascular disease in 184 cities in China from 2014 to 2017.
- City-specific associations between temperature variability and hospital admissions for cardiovascular disease were estimated with overdispersed Poisson models. Random-effects meta-analyses were applied to obtain national and regional average associations.
- Our results showed that temperature variability was associated with increased hospital admissions for ischemic heart disease, heart failure, heart rhythm disturbances, and ischemic stroke.

**What do these findings mean?:**

- To our knowledge, this is the first national study in China to examine the associations between temperature variability and daily hospital admissions for cause-specific cardiovascular disease.
- Our findings suggested that general practitioners, cardiologists, and public health officials should consider the detrimental effects of temperature variability in their mitigation strategies.

## Introduction

Cardiovascular disease is a major cause of death and disability worldwide [1,2]. Climate change has been considered potentially the greatest threat to human health of the 21st century [3–5]. In the past few decades, increasing epidemiological studies have reported associations of mortality and morbidity from cardiovascular disease with ambient temperature [6,7]. However, daily mean temperature failed to reflect intra- and interday variations of temperature. Temperature variability is an important meteorological indicator reflecting climate changes, such as rapid temperature fluctuations within a certain period (e.g., intra- and interday changes in temperature) [8,9]. It may also pose a major danger to human health. To date, however, scientific evidence is still inadequate regarding the association between daily temperature variation and cardiovascular disease morbidity, especially in developing countries. Quantifying the effect of temperature variability on cardiovascular disease has major public health implications, particularly in the context of increasing climate change and unstable weather patterns [10,11].

In China, climate change has emerged as a significant public health problem [12]. Recently, several studies have examined the association between temperature variability and daily mortality [13–15]. However, a common limitation of these studies was their focus on a single city or several sites in a local area. Previous studies have demonstrated that climate conditions, sociodemographic status, and locations of study sites could modify the health effects of temperature variability [8,16]. China has a vast territory and varied terrain, as well as a great diversity of climates, including tropical in southern regions to subarctic in the extreme north. Therefore, the associations between short-term temperature variability and cause-specific cardiovascular disease morbidity remain unclear at the national level. A national analysis is important to comprehensively assess the health effects of temperature variation in China.

In this study, we designed a national time-series study to examine the short-term associations between temperature variability and hospital admissions of cause-specific cardiovascular disease for 2014–2017 in urban China as well as to investigate individual-level and city-level potential effect modifiers.

## Methods

### Study sites

A total of 184 cities were included in this analysis. The inclusion of these cities was on the basis of the availability of both weather and health data. Cities with records of less than 1 year were excluded due to the feasibility of model fit. Individuals’ detailed information of the disease diagnosis was required to identify the cardiovascular admissions. Cities with no information on International Classification of Diseases (ICD) code or those whose text of disease diagnosis cannot be classified as categories of cardiovascular disease were also excluded. S1 Fig presents the locations of the 184 cities, representing a geographic distribution across China.

### Data collection

There are three main health insurance programs in China: the Urban Employee Basic Medical Insurance (UEBMI) for urban working and retired employees, the Urban Residence Basic Medical Insurance for urban residents without formal employment, and the New Rural Cooperative Medical Scheme for rural residents. These three programs covered more than 92% of the population by 2011 [17].

Daily counts of hospital admissions within each city were obtained from the database of UEBMI. In 2016, there were 0.28 billion beneficiaries in 31 provincial administrative regions, representing approximately one-fifth of the total population in Mainland China. The size of this population allows for investigation of cause-specific cardiovascular diseases that have been associated with temperature variability [18]. The number of city residents, people enrolled in the database, and coverage rate of the population by UEBMI are presented in S1 Table. The number of city’s residents is according to the 2010 census. Among the 184 cities, Jiayuguan is the smallest city with a population of 23.19 thousand, and Chongqing is the largest city with a population of 28.8 million. The medical information recorded on the database includes age, sex, date of medical service, diagnosis, and financial cost. Hospital admissions for each health condition were identified based on the primary diagnoses (text of disease diagnosis or ICD code). Cardiovascular admissions included ischemic heart disease (ICD-10 codes I20–I25), heart failure (I50), heart rhythm disturbances (I47–I49), and ischemic stroke (I63) [19]. Admissions aged <18 years were too few and therefore were not included. Because the data used for this study were collected for administrative purpose without any individual identifiers, this study was exempted from Institutional Review Board approval by the Ethics Committee of Peking University Health Science Center, Beijing, China. The need for informed consent was also waived by the Institutional Review Board.

Data on temperature and relative humidity for each city were acquired from the China Meteorological Data Sharing Service Center. There are one to three monitoring stations in each city. We calculated daily maximum, minimum, and mean temperatures and mean relative humidity by averaging all valid monitoring measurements in each city. During the study period, the missing rate of temperature and relative humidity was only 0.25%. To control for the potential confounding effects of ambient air pollution, we obtained data on fine particulate matter (PM_2.5_), NO_2_, and SO_2_ concentrations for each city from the National Air Pollution Monitoring System. There are 1 to 17 monitors in each city. We calculated daily mean levels of PM_2.5_, NO_2_, and SO_2_ by averaging all valid monitoring measurements in each city. During the study period, the missing rates were 0.66% for PM_2.5_, 0.15% for NO_2_, and 0.15% for SO_2_, respectively. Days with missing information on meteorological variables or the air pollution data were excluded from analysis.

### Exposure definition

In this study, we employed a composite indicator of intraday and interday temperature variability to assess the effects of temperature variation on daily hospital admissions. The definition of the temperature variability indicator has been described previously [8]. Temperature variability index was calculated from the standard deviation (SD) of daily minimum and maximum temperatures over the exposure days. For example, temperature variability for the preceding 2 days’ exposure (TV_0–1_) was calculated as SD (MinTemp_lag0_, MaxTemp_lag0_, MinTemp_lag1_, MaxTemp_lag1_) such that MinTemp_lagi_ and MaxTemp_lagi_ represent the minimum and the maximum temperature for the preceding day i; temperature variability for the preceding 3 days’ exposure was calculated by TV_0–2_ = SD (MinTemp_lag0_, MaxTemp_lag0_, MinTemp_lag1_, MaxTemp_lag1_, MinTemp_lag2_, MaxTemp_lag2_), and so on. This index could account for both intraday and interday temperature variability, as well as the delayed effects of temperature variation [8].

### Statistical analysis

The associations between temperature variability and daily hospital admissions for cardiovascular disease were estimated by a commonly used two-stage approach [8,9]. The method and the model used in this study were designed before the analyses were conducted, and the prespecified statistical analysis plan was present in Supporting Information (S1 Appendix). In the first stage, quasi-Poisson regression models were applied for estimating city-specific estimates of hospital admissions associated with temperature variability exposure. Previous studies have observed that mortality risk is almost linearly elevated with increases in temperature variability [8,9,20]. We then designed a linear term for temperature variability to be included in the model. Furthermore, consistent with previous studies [8,9,18], several confounders were included in the model, as follows:

1. A natural cubic spline of calendar time with 7 degrees of freedom (df) per year to adjust for unmeasured time trends longer than 2 months in hospital admissions
2. A natural cubic spline of 3-day moving average relative humidity with 3 df
3. Indicator variables for day of the week and public holiday
4. To adjust for the confounding effect of daily mean temperature, a distributed lag nonlinear model (DLNM) for daily mean temperature was also included in the model [8,9,21].

Specifically, we used two natural cubic spline functions with 4 df, respectively, for daily mean temperature and lag over time up to 21 days to accommodate the nonlinear and lagged effects of ambient temperature. The selection of 21 days was based on the evidence that the effect of cold temperature was delayed and lasted for several weeks, while the effect of hot temperature was acute and generally presented within 1 week [22,23]. Three internal knots were placed at equally spaced temperature percentiles (25th, 50th, and 75th). Consequently, the model was as shown below:

$$Log\left[E\left(Y_{t}\right)\right]=\alpha +\beta \;\left(temperature\;variability\right)+\gamma \;Temperature+day\;of\;the\;week+public\;holidays+ns\left(calendar\;time,\;df=7/per\;year\right)+ns\left(relative\;humidity,\;df=3\right)$$

such that E(Y_t_) is the expected count of admissions on day t, *β* represents the log-relative risk of admission associated with a unit increase of temperature variability, “Temperature” indicates a two-dimensional cross-basis matrix produced by DLNM, and *ns*() indicates natural cubic spline function. Public holidays and day of the week were included in the model as indicator variables, and “relative humidity” indicates 3-day moving average relative humidity. In the second stage, the national and regional average estimates of the associations between temperature variability and hospital admissions were obtained by combining city-specific estimates using a random-effects meta-analysis. *I*^2^ statistics were calculated to examine the heterogeneity of city-specific estimates. We conducted separate analyses for temperature variability during different exposure days from TV_0–1_ to TV_0–7_ [8,18].

To quantify the uncertainty in heterogeneity estimates, we also calculated 95% confidence interval (CI) for *I*^2^ [24]. To assess the shape association between temperature variability and the outcome, we used the same approach as the “Air Pollution and Health: A European Approach” (APHEA) project and a recent multicity analysis on the association between temperature variability and mortality [9,25,26]. A natural cubic spline was applied. We also investigated potential effect modifiers of the association between temperature variability and daily hospital admissions for cardiovascular disease. We conducted stratified analyses by age, sex, and geographical region. Due to the regional differences in weather conditions, geography, and culture, the 184 cities were grouped into south and north regions [27,28]. The *P* values for difference in estimates between the strata were obtained via Z-test [29].

In addition, we explored the confounding influence of air pollutants or potential effect modifications by cities’ characteristics. To assess the confounding effect of air pollutants, the model was further adjusted for the linear terms of daily PM_2.5_, NO_2_, and SO_2_ concentrations. To evaluate the potential effect modifications by cities’ characteristics, city-specific relative risk (and CIs) as the outcome were meta-regressed on each continuous variable of city characteristics. City-level characteristics included annual-average temperature variability levels, temperature and relative humidity, gross domestic product (GDP) per capita, and coverage of population by UEBMI. We performed random-effects meta-regression model using city-level estimations from the first stage as: y_i_ = α + βx_i_ + μ_i_ + e_i_ such that y_i_ is the city-specific estimation, x_i_ is the city-level characteristic variable, μ_i_ is a normal error term with known SDs within-city standard error that may vary across units, and e_i_ is a normal error with variance to be estimated, assumed equal across units [30]. The model was implemented by the “metareg” function in STATA.

### Sensitivity analysis

Sensitivity analyses were conducted by changing the maximum lag of temperature from 21 to 28 days, df for daily mean temperature (3–6), and df for time trend (6–8 per year), respectively. We also assessed the robustness of the results after controlling for daily minimum or maximum temperature instead of daily mean temperature. In addition, we used penalized spline functions for time trend and meteorological variables. Finally, we reported estimated effects using interquartile range (IQR) increase of temperature variability.

Statistical analyses were performed in R software version 3.2.2 (R Foundation for Statistical Computing, Vienna, Austria) and STATA software version 12 (StataCorp, College Station, TX). The results were presented as percentage change and 95% CI in hospital admissions per 1-°C increase in temperature variability. Percentage change equals relative risk minus 1 and then multiplied by 100.

## Results

Table 1 presents the demographic characteristics of people enrolled in the UEBMI in the 184 Chinese cities in 2017. Of the 184 cities, 94 cities were located in south region and 90 cities were in the north. Overall, there were 54.4% male patients and 4.9% patients aged ≥75 years. The sex and age distributions of participants in the south versus north regions were similar. For the 184 cities, there were 8.0 million hospital admissions for cardiovascular disease from January 1, 2014, through December 31, 2017. S2 and S3 Tables present city-specific summary statistics on annual-average hospital admissions for cause-specific cardiovascular disease, weather variables, temperature variability, and air pollutants. Summary statistics of daily hospital admissions for cardiovascular disease, weather conditions, temperature variability, and air pollutants are presented in Table 2. On average, we recorded 44 cardiovascular admissions per day across the cities, with a range from 1 to 302. The annual-average daily mean temperature was 14.4°C, with a range from −0.2°C to 24.4°C. The annual-average TV_0–1_ was 5.7°C, ranging from 2.9°C to 9.3°C. The distributions of temperature variability were similar at different exposure days (TV_0–1_ to TV_0–7_). The distribution of daily temperature variability at different exposure days is presented in S4 Table.

**Table 1:** Demographic characteristics of people enrolled in the UEBMI in the 184 Chinese cities in 2017.

Variable	Nationwide	South	North
**Total**	197,230,556	127,263,223	69,967,333
**Sex**			
Male (%)	107,209,773 (54.4)	68,600,413 (53.9)	38,609,360 (55.2)
Female (%)	72,507,689 (36.8)	58,662,810 (46.1)	31,357,973 (44.8)
**Age (year)**			
18–64 (%)	172,616,807 (87.5)	113,036,386 (88.8)	59,580,421 (85.2)
≥65–74 (%)	14,553,516 (7.4)	8,376,202 (6.6)	6,177,314 (8.8)
≥75 (%)	9,645,159 (4.9)	5,448,893 (4.3)	4,196,266 (6.0)

**Abbreviation:** UEBMI, Urban Employee Basic Medical Insurance.

**Table 2:** Summary statistics of daily hospital admissions for cardiovascular disease, weather conditions, air pollutants, and temperature variability in 184 Chinese cities, 2014–2017.

Variables	Mean	Range
**Cardiovascular disease**	44	1 to 302
**Relative humidity (%)**	68	33 to 92
**Temperature (°C)**	14.4	−0.2 to 24.4
**PM**_**2.5**_**, μg/m**^**3**^	50.4	14.8 to 155.3
**NO**_**2**_**, μg/m**^**3**^	32.0	12.8 to 60.3
**SO**_**2**_**, μg/m**^**3**^	26.4	2.3 to 93.3
**Temperature variability**		
TV_0–1_ (°C)	5.7	2.9 to 9.3
TV_0–2_ (°C)	5.6	2.8 to 8.9
TV_0–3_ (°C)	5.6	2.8 to 8.8
TV_0–4_ (°C)	5.6	2.8 to 8.7
TV_0–5_ (°C)	5.6	2.8 to 8.6
TV_0–6_ (°C)	5.6	2.8 to 8.6
TV_0–7_ (°C)	5.6	2.8 to 8.6

**Abbreviations:** PM_2.5_, fine particulate matter; TV_0-1_, temperature variability at 0–1 days.

**Fig 1:**
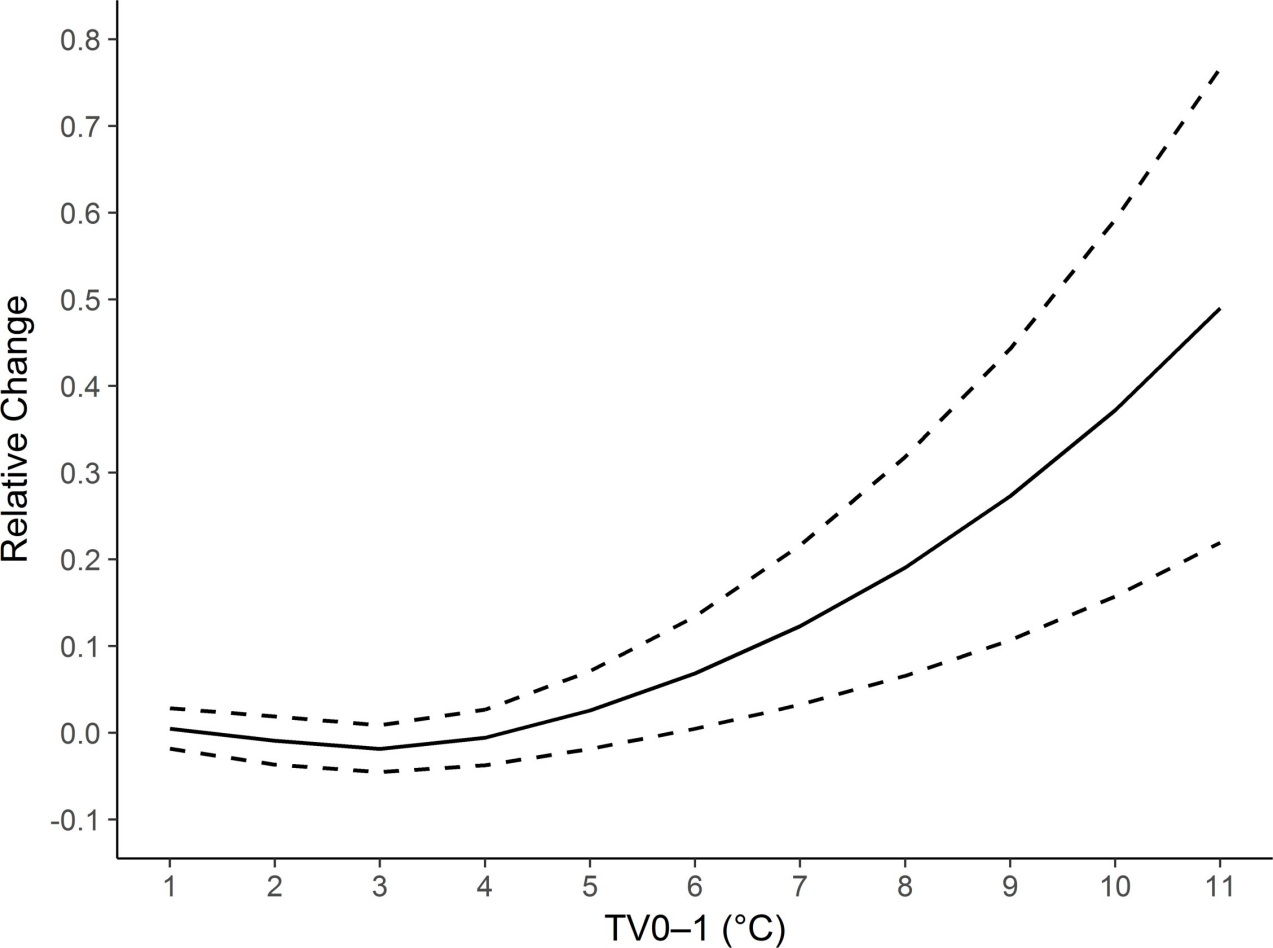
National-average exposure-response association curve between TV_0–1_ and daily hospital admissions for cardiovascular disease in 184 cities in China, 2014–2017. TV_0–1_, temperature variability at 0–1 days.

Fig 1 presents the national-average exposure-response curve between TV_0–1_ and hospital admissions for cardiovascular disease. We noted a broadly linear association. The curve was relatively flat at low levels (<3°C) and increased rapidly at levels greater than 3°C.

**Fig 2:**
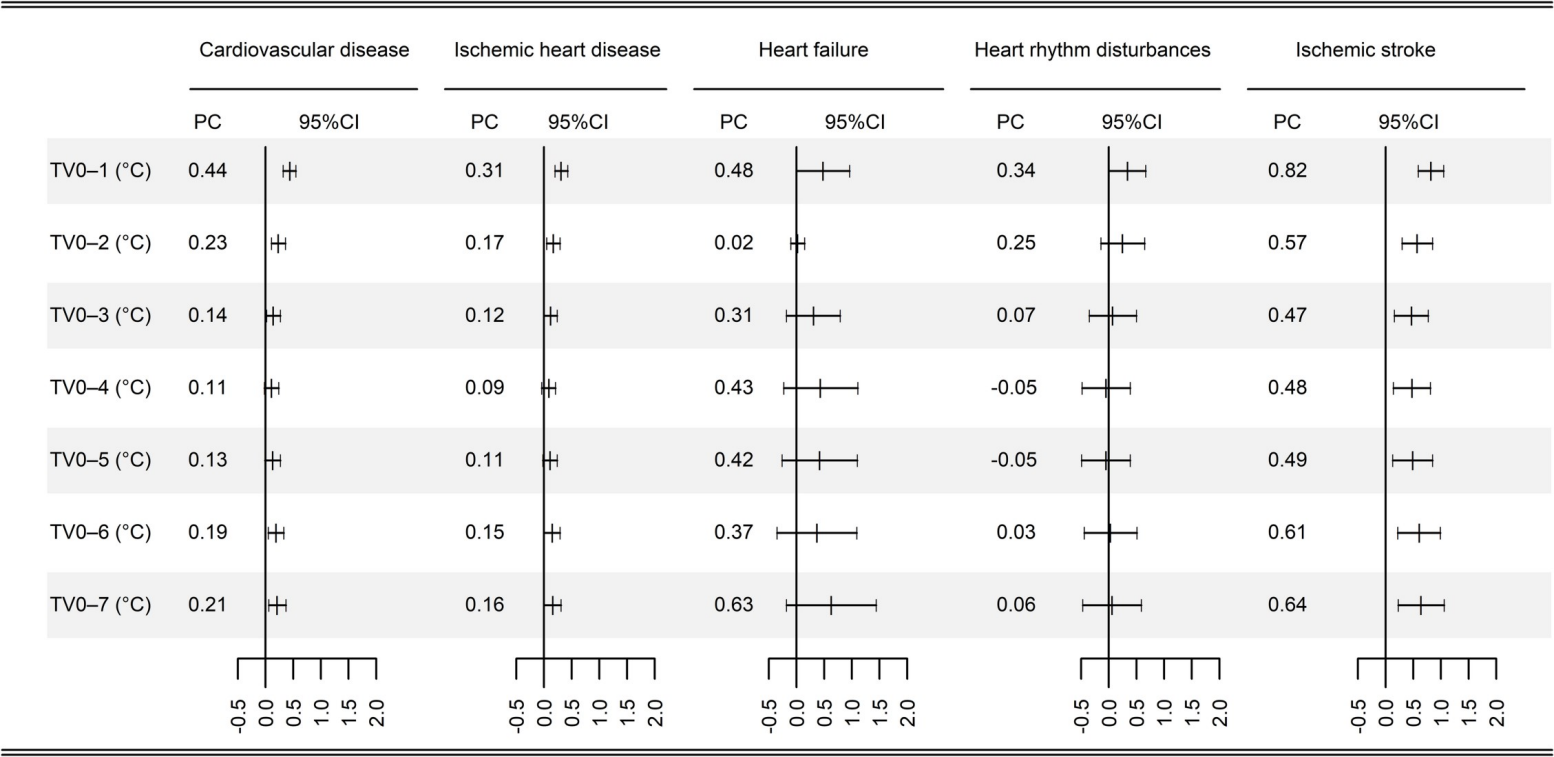
National-average PC with 95% CI in daily hospital admissions for cause-specific cardiovascular disease per 1-°C increase in temperature variability at different exposure days in 184 Chinese cities, 2014–2017. CI, confidence interval; PC, percentage change; TV_0–1_, temperature variability at 0–1 days.

Fig 2 summarizes the national-average percentage changes in hospital admissions for cardiovascular diseases associated with 1-°C increase in temperature variability at different exposure days. We observed weak or moderate between-city heterogeneity for the associations between temperature variability and hospital admissions for cardiovascular disease (*I*^2^ = 47.8%; 95% CI 38.0%–56.0%), ischemic heart disease (*I*^2^ = 36.5%; 24.1%–47.0%), heart failure (*I*^2^ = 3.8%; 0%–15.1%), heart rhythm disturbances (*I*^2^ = 21.2%; 5.1%–34.5%), and ischemic stroke (*I*^2^ = 51.4%; 42.1%–59.3%). The largest estimates were observed at exposure 0–1 days for all health outcomes except heart failure. For a 1-°C increase in TV_0–1_, we observed significant increases of 0.44% (0.32%–0.55%) in hospital admissions for cardiovascular disease, 0.31% (0.20%–0.43%) for ischemic heart disease, 0.48% (0.01%–0.96%) for heart failure, 0.34% (0.01%–0.67%) for heart rhythm disturbances, and 0.83% (0.60%–1.06%) for ischemic stroke.

**Fig 3:**
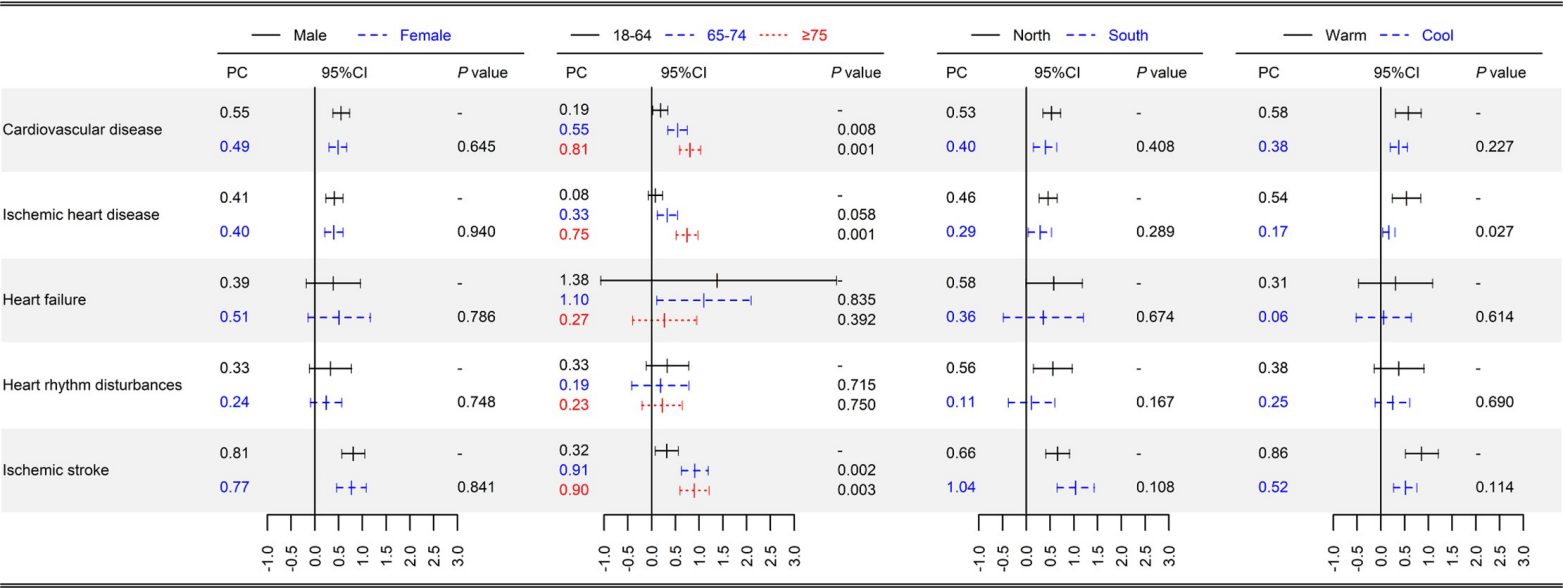
National-average PC with 95% CI in daily hospital admissions for cause-specific cardiovascular disease per 1-°C increase in TV_0–1_, stratified by sex, age, and geographical region. CI, confidence interval. CI, confidence interval; PC, percentage change; TV_0–1_, temperature variability at 0–1 days.

Fig 3 shows the results of the stratified analyses. The associations between TV_0–1_ and cardiovascular disease varied by age. For cardiovascular disease, the estimates were larger in people aged 65–74 years and ≥75 years than in people aged 18–64 years (0.81 [0.59–1.03] in aged ≥75 versus 0.55 [0.34–0.75] in aged 65–74 versus 0.19 [0.03–0.34] in aged 18–64 group [*P* < 0.05]), similar to in ischemic heart disease and ischemic stroke (0.75 [0.52–0.98] in aged ≥75 versus 0.08 [−0.07 to 0.23] in aged 18–64 group [*P* < 0.001] for ischemic heart disease and 0.90 [0.60–1.21] in aged ≥75 versus 0.32 [0.08–0.56] in aged 18–64 group [*P* = 0.003] for ischemic stroke). The estimates were broadly similar between males and females (Fig 3, all *P* > 0.05), between northern and southern regions (Fig 3, all *P* > 0.05), and among different climate temperature groups (S5 Table). We divided the cities into four groups based on the quantiles of their annual-average temperatures: cold area (≤25th), moderate cold area (25th–50th), moderate hot area (50th–75th), and hot area (>75th). We observed consistent significant associations in different climate areas. A 1-°C increase in TV_0–1_ corresponded to a 0.38% (0.15%–0.62%), 0.60% (0.34%–0.86%), 0.43% (0.13%–0.73%), and 0.55% (0.18%–0.93%) higher cardiovascular disease admissions in cold, moderate cold, moderate hot, and hot areas, respectively. The estimates for all health conditions were higher in warm season than in cool season, although the difference was only significant for ischemic heart disease (*P* = 0.027).

The associations between TV_0–1_ and cardiovascular disease after further adjustment of air pollutants are shown in Table 3. The estimates decreased but remained significant after adjusting for PM_2.5_ (0.39% change; 0.22%–0.56%), NO_2_ (0.19% change; 0%–0.38%), and SO_2_ (0.30% change; 0.13%–0.47%). Table 4 presents the results of effect modification by city-level characteristics. Annual-average temperature variability, temperature and relative humidity levels, GDP per capita, and coverage rate of population by UEBMI did not significantly modify the associations between TV_0–1_ and hospital admissions for cardiovascular disease (*P* > 0.05).

**Table 3:** Results on the associations between TV_0–1_ and hospital admissions for cardiovascular disease after controlling for the air pollutants.

Variables	PC per 1-°C increase in TV_0–1_	95% CI	*P*
**Adjust for PM**_**2.5**_	0.39	0.22–0.56	<0.001
**Adjust for NO**_**2**_	0.19	0–0.38	0.045
**Adjust for SO**_**2**_	0.30	0.13–0.47	<0.001

**Abbreviations:** CI, confidence interval; PC, percentage change; PM_2.5_, fine particulate matter; TV_0–1_, temperature variability at 0–1 days.

**Table 4:** Meta-regression results of the modification effects of city-level characteristics on the associations between TV_0–1_ and hospital admissions for cardiovascular disease in 184 Chinese cities, 2014–2017.

Variables	PC per 1-°C increase in TV_0–1_	95% CI	*P*
Temperature variability (°C)	0.006	−0.112 to 0.125	0.916
Temperature (°C)	0.009	−0.024 to 0.041	0.602
Relative humidity (%)	0.001	−0.014 to 0.015	0.955
GDP per capita	0.025	−0.048 to 0.098	0.502
Coverage of population (%)	−0.002	−0.011 to 0.008	0.724

**Abbreviations:** CI, confidence interval; GDP, gross domestic product; PC, percentage change; TV_0–1_, temperature variability at 0–1 days.

Table 5 shows the results of sensitivity analyses. The association between TV_0–1_ and cardiovascular disease was insensitive to changing maximum lag of temperature (28 days) and df for time (6–8 per year) and temperature (3–6). The estimate slightly changed when using penalized spline functions in the model (0.48% change; 0.31%–0.65%). The association remained after adjustment of daily minimum and maximum temperature. We also applied a threshold approach to estimate the association and found that a 1-°C increase in TV_0–1_ was associated with a 0.48% (0.33%–0.64%) increase in hospital admissions for cardiovascular disease if temperature variability > 3°C. The estimates for associations between temperature variability and cause-specific cardiovascular diseases are presented in S6 Table. The estimated effects in IQR increase of TV_0–1_ (3.1°C) are presented in S7 Table.

**Table 5:** Results of sensitivity analyses on the associations between TV_0–1_ and hospital admissions for cardiovascular disease in 184 Chinese cities, 2014–2017.

Variables	PC per 1-°C increase in TV_0–1_	95% CI	*P*
**df for calendar time**			
6	0.46	0.31–0.62	<0.001
7	0.44	0.32–0.55	<0.001
8	0.43	0.28–0.57	<0.001
**df for temperature**			
3	0.50	0.34–0.66	<0.001
4	0.44	0.32–0.55	<0.001
5	0.50	0.34–0.66	<0.001
6	0.50	0.34–0.65	<0.001
**Maximum lag of temperature (28 days)**	0.45	0.28–0.61	<0.001
**Penalized spline function**	0.48	0.31–0.65	<0.001
**Adjust for daily minimum temperature**	0.30	0.13–0.47	<0.001
**Adjust for daily maximum temperature**	0.53	0.37–0.69	<0.001
**Association if temperature variability > 3°C**	0.48	0.33–0.64	<0.001

**Abbreviations:** CI, confidence interval; df, degrees of freedom; PC, percentage change; TV_0–1_, temperature variability at 0–1 days.

## Discussion

In this national study, we found that short-term temperature variability exposure was associated with increased hospital admissions for cardiovascular disease, after controlling for daily mean temperature. The associations were more evident in the elderly but did not vary substantially by climates (annual-average temperature variability, temperature and relative humidity levels), demographic characteristics (sex and GDP per capita), and geographical region. These associations were robust to the adjustment of air pollutants (PM_2.5_, NO_2_, and SO_2_).

A number of epidemiological studies have estimated the cardiovascular disease risk in association with intraday or interday temperature variation separately [20,31,32]. However, only capturing the impact of intraday or interday temperature variation might underestimate the true effect of temperature variability [8,18]. Few studies to date have considered both intra- and interday temperature variability in an attempt to comprehensively evaluate the acute effect of temperature variability on cardiovascular disease. A pooled estimate of 12 counties across Hubei Province in China reported a 1.72% (0.69%–2.76%) increase in cardiovascular disease mortality in relation to an increment of 1°C in TV_0–7_ [15]. A recent study with 31 major Chinese cities demonstrated that a 1-°C increase in TV_0–7_ was associated with a 0.60% (0.25%–0.94%) increase in cardiovascular disease mortality [18]. However, air pollution, an important environmental risk factor for cardiovascular disease, was not controlled for in these two studies. Therefore, the two studies might have overestimated the effect of temperature variability on cardiovascular disease. In this study, the estimates substantially decreased when controlling for PM_2.5_, NO_2_, and SO_2,_ which was consistent with a recent study that reported a weakened association of temperature variability with cardiovascular disease mortality after adjusting for air pollution [14]. Specifically, to further explore the modification effect of NO_2_ on the association between TV_0–1_ and cardiovascular disease, we divided the cities into three groups based on the tertiles of their annual average NO_2_ levels. The association was stronger in cities with higher annual average NO_2_ levels (S8 Table). The association between temperature variability and cardiovascular admissions was attenuated but remained significant after adjustment of air pollutants, suggesting that air pollution is a confounder of the association.

Overall, we found significant associations between temperature variability and hospital admissions for cause-specific cardiovascular disease in China, and these associations were strongest at lag 0–1 days. Several studies have reported stronger effects of daily temperature variation on cardiovascular disease mortality at longer exposure days [14,15]. Differences in these findings might be attributable to different health outcomes. In this study, we utilized hospital admission data rather death data. Cardiovascular disease admissions can differ markedly from death events from cardiovascular disease by volume, severity, and demographics. In addition, cardiovascular disease admissions are more sensitive to short-term exposure to environmental factors and therefore could better explore the temporal pattern between temperature variability and clinical presentation of cardiovascular disease [33]. A recent multicountry study reported that the highest estimates of temperature-variability–mortality appeared at different exposure days in different countries [8]. Therefore, it was plausible to hypothesize that study sites’ characteristics, such as climates, geographical region, and population susceptibility, might also modify the lag pattern between temperature variability exposure and cardiovascular disease.

We observed a broadly linear exposure-response curve between temperature variability and cardiovascular disease, which is consistent with previous findings [8,9,20]. We also noted that the curve was relatively flat at low levels, in line with the curve for the association between temperature variability and mortality observed in Melbourne, Australia [9]. Human core body temperature is normally maintained within a narrow range via heat exchange with the surrounding environments. People may be able to adapt well to changes in temperature when they are relatively minor. However, people may have difficulty with thermoregulation to sudden, dramatic temperature changes not only physiologically but also in terms of behavioral patterns [34–36]. Previous studies have demonstrated that unstable temperatures could alter heart rate, blood pressure, blood cholesterol level, inflammatory reaction, and immune function [37–40]. These physiological changes may trigger cardiovascular disease [41].

In line with previous studies [15,18,20,42], we found that the elderly have a higher risk of cardiovascular disease during days with great temperature variation compared with the younger group. The decline of thermoregulatory function occurs naturally in aging adults [43], which might partly explain the increased vulnerability to temperature variability in the elderly. In addition, elderly people generally have a higher prevalence of chronic health conditions, which might also contribute to the increased vulnerability. China is experiencing rapid population aging, therefore the cardiovascular disease burden attributed to temperature variability is expected to increase. Moreover, unlike other risk factors, temperature variability exposure is ubiquitous. As a result, temperature-variability–related burden of cardiovascular disease should be an increasingly serious environmental health issue in China.

We observed weak or moderate between-city heterogeneity for the associations between temperature variability and hospital admissions for heart failure, heart rhythm disturbances, and ischemic heart disease, indicating that the effects of temperature variability on these health outcomes might not vary substantially by geographical and demographic characteristics in China. This hypothesis is further supported by the absence of evidence for effects modification by city’s characteristics, as shown in the meta-regression analyses. However, we observed relatively stronger heterogeneity of city-specific estimates for ischemic stroke. This might be attributable to the substantial regional variation in baseline incidence of ischemic stroke. A recent nationwide population-based study in China demonstrated significant regional variations in annual incidence rate of stroke [44]. City-specific characteristics, such as socio-demography, chronic diseases burden, or lifestyles, might also be responsible for the heterogeneity. Further investigation with more detailed information on individuals’ lifestyle risk factors is required to confirm this finding.

This study has several strengths. We analyzed multicity data using a uniform statistical method, thus enhancing internal comparison of results across these cities, as well as external comparison with other studies. Our study should provide more representative estimates of the associations between temperature variability and hospital admissions for cause-specific cardiovascular disease. In addition, we included 184 cities involving great diversity in temperature variability levels, meteorological conditions, and geography, enabling us to evaluate the potential effect modification by these characteristics. Finally, a range of analyses were performed to test the robustness of the findings, including additional adjustment of air pollution.

Several limitations should also be acknowledged. First, we used temperature data from fixed monitors as a surrogate of personal exposure, which can cause exposure measurement errors. This kind of nondifferential error is likely to bias the estimates downward [45]. Second, misclassification bias caused by diagnostic error should be considered when interpreting the results. However, this error is likely to be random and typically reduce the precision of the estimates and leads to underestimation of estimates [46]. Finally, children and adolescents (age group 0–18) were not included in this assessment because UEBMI is for the urban working and retired employees, though young children may have more difficulty in regulating their temperature, similarly to the elderly.

In conclusion, this national study in 184 Chinese cities, which is, to our knowledge, the largest study worldwide to date, provides evidence of a relationship between daily temperature variation and increased hospital admissions for cardiovascular disease. The findings have significant implications for general practitioners, cardiologists, and public health officials.

## Supporting information

S1 STROBE Checklist STROBE checklist. (DOC)

S1 Table City-specific number of people enrolled in the UEBMI in 2017, the number of residents, and the coverage rate based on the UEBMI in 184 Chinese cities. UEBMI, Urban Employee Basic Medical Insurance.
(DOCX)

S2 Table Summary statistics on annual-average hospital admissions for cause-specific cardiovascular disease in 184 Chinese cities, 2014–2017. (DOCX)

S3 Table Summary statistics on annual-average TV_0–1_, weather conditions, and air pollutants in 184 Chinese cities, 2014–2017. TV_0–1_, temperature variability at 0–1 days.
(DOCX)

S4 Table The distribution of daily temperature variability at different exposure days in 184 cities in China, 2014–2017. (DOCX)

S5 Table National-average PC with 95% CI in daily hospital admissions for cardiovascular disease associated with a 1-°C increase in TV_0–1_ in 184 Chinese cities by climate region, 2014–2017. CI, confidence interval; PC, percentage change; TV_0–1_, temperature variability at 0–1 days.
(DOCX)

S6 Table National-average PC with 95% CI in daily hospital admissions for cause-specific cardiovascular disease per 1-°C increase in TV_0–1_ if temperature variability > 3°C in 184 Chinese cities, 2014–2017. CI, confidence interval; PC, percentage change; TV_0–1_, temperature variability at 0–1 days.
(DOCX)

S7 Table National-average PC with 95% CI in daily hospital admissions for cause-specific cardiovascular disease per IQR increase in TV_0–1_ (3.1°C) in 184 Chinese cities, 2014–2017. CI, confidence interval; IQR, interquartile range; PC, percentage change; TV_0–1_, temperature variability at 0–1 days.
(DOCX)

S8 Table National-average PC with 95% CI in daily hospital admissions for cardiovascular disease associated with a 1-°C increase in TV_0–1_ in 184 Chinese cities, 2014–2017, classified by city-specific annual average NO_2_ levels. CI, confidence interval; PC, percentage change; TV_0–1_, temperature variability at 0–1 days.
(DOCX)

S1 Fig The locations of 184 cities analyzed in this study. The most populous city in each province was marked by name.
(TIF)

S1 Appendix Statistical analysis plan. (DOCX)

## Abbreviations

CI: confidence interval
df: degrees of freedom
DLNM: distributed lag nonlinear model
GDP: gross domestic product
ICD: International Classification of Diseases
IQR: interquartile range
PC: percentage change
PM_2.5_: fine particulate matter
SD: standard deviation
TV_0–1_: temperature variability at 0–1 days
UEBMI: Urban Employee Basic Medical Insurance
