# Supplementary material for: Association between temperature variability and daily hospital admissions for cause-specific cardiovascular disease in urban China: A national time-series study
Source: PLoS Med. 2019 Jan 28;16(1):e1002738. doi: 10.1371/journal.pmed.1002738 (PMC6349307; doi:10.1371/journal.pmed.1002738)
Supplement: S1 Table — UEBMI, Urban Employee Basic Medical Insurance. (DOCX) [file pmed.1002738.s002.docx]

**S1 Table:** City-specific number of people enrolled in the Urban Employee Basic Medical Insurance (UEBMI) in 2017, the number of residents, and the coverage rate based on the UEBMI in 184 Chinese cities.

| City | Number of people enrolled in the UEBMI in 2017 (thousand) | Number of city's residents (thousand)^*^ | Coverage rate of the population (%) |
| --- | --- | --- | --- |
| An'shan | 1169.735 | 3645.9 | 32.08 |
| Anyang | 639.594 | 5172.8 | 12.36 |
| Baicheng | 238.969 | 2033.1 | 11.75 |
| Baise | 352.491 | 3466.8 | 10.17 |
| Baiyin | 267.624 | 1708.8 | 15.66 |
| Baoji | 578.582 | 3716.7 | 15.57 |
| Baoshan | 162.638 | 2506 | 6.49 |
| Baotou | 881.293 | 2650.3 | 33.25 |
| Bayannur | 258.573 | 1669.9 | 15.48 |
| Bazhong | 215.918 | 3283.7 | 6.58 |
| Beihai | 248.703 | 1539.3 | 16.16 |
| Bengbu | 518.759 | 3164 | 16.40 |
| Benxi | 783.511 | 1709.5 | 45.83 |
| Binzhou | 643.217 | 3748.5 | 17.16 |
| Bozhou | 293.575 | 4851 | 6.05 |
| Changde | 515.205 | 5717.2 | 9.01 |
| Changsha | 1226.816 | 7044.1 | 17.42 |
| Changzhi | 649.839 | 3334.6 | 19.49 |
| Changzhou | 2082.949 | 4592 | 45.36 |
| Chaoyang | 496.376 | 3044.6 | 16.30 |
| Chengde | 543.004 | 3473.2 | 15.63 |
| Chengdu | 8084.426 | 14047.6 | 57.55 |
| Chenzhou | 483.704 | 4581.8 | 10.56 |
| Chifeng | 652.651 | 4341.2 | 15.03 |
| Chizhou | 156.549 | 1403 | 11.16 |
| Chongqing | 7033.574 | 28846.2 | 24.38 |
| Chongzuo | 186.098 | 1194.3 | 15.58 |
| Chuzhou | 485.798 | 3938 | 12.34 |
| Dalian | 4739.327 | 6690.4 | 70.84 |
| Dandong | 842.188 | 2444.7 | 34.45 |
| Datong | 694.392 | 3318.1 | 20.93 |
| Daxinganling | 114.424 | 511.6 | 22.37 |
| Dazhou | 430.925 | 5468.1 | 7.88 |
| Dezhou | 699.408 | 5568.2 | 12.56 |
| Dongying | 534.658 | 2035.3 | 26.27 |
| Erdos | 438.985 | 1940.7 | 22.62 |
| Fangchenggang | 131.863 | 866.9 | 15.21 |
| Fushun | 920.459 | 2138.1 | 43.05 |
| Guangyuan | 392.252 | 2484.1 | 15.79 |
| Guangzhou | 12970.73 | 12700.8 | 102.13 |
| Guigang | 271.472 | 4118.8 | 6.59 |
| Guyuan | 115.925 | 1228.2 | 9.44 |
| Haikou | 748.422 | 2046.1 | 36.58 |
| Handan | 1119.457 | 9174.6 | 12.20 |
| Hangzhou | 6671.225 | 8700.4 | 76.68 |
| Hefei | 2263.288 | 5702 | 39.69 |
| Hegang | 128.822 | 1058.7 | 12.17 |
| Heihe | 114.306 | 1633.9 | 7.00 |
| Hengshui | 382.178 | 4340.8 | 8.80 |
| Hengyang | 850.294 | 7141.5 | 11.91 |
| Hetian | 181.223 | 2014.3 | 9.00 |
| Heze | 705.687 | 8287.8 | 8.51 |
| Hezhou | 178.734 | 1954.1 | 9.15 |
| Hinggan League | 229.829 | 1613.3 | 14.25 |
| Hohhot | 755.669 | 2866.6 | 26.36 |
| Huai'an | 924.739 | 4799.9 | 19.27 |
| Huaibei | 438.918 | 2114 | 20.76 |
| Huaihua | 402.953 | 4741.9 | 8.50 |
| Huainan | 530.365 | 2334 | 22.72 |
| Huangshan | 215.696 | 1359 | 15.87 |
| Huludao | 660.431 | 2623.5 | 25.17 |
| Hulunbeier | 708.438 | 2549.3 | 27.79 |
| Jiaxing | 2526.202 | 4501.7 | 56.12 |
| Jiayuguan | 99.73 | 231.9 | 43.01 |
| Jilin | 938.347 | 4414.7 | 21.26 |
| Jinan | 3198.532 | 6814 | 46.94 |
| Jincheng | 524.833 | 2279.2 | 23.03 |
| Jinhua | 1639.551 | 5361.6 | 30.58 |
| Jining | 1313.794 | 8081.9 | 16.26 |
| Jinzhong | 644.623 | 3249.4 | 19.84 |
| Jinzhou | 838.432 | 3126.4 | 26.82 |
| Jixi | 284.222 | 1862.1 | 15.26 |
| Karamay | 77.782 | 391 | 19.89 |
| Kashi | 375.495 | 3979.3 | 9.44 |
| Kiamusze | 437.396 | 2552.1 | 17.14 |
| Kunming | 1715.482 | 6432 | 26.67 |
| Laibin | 194.652 | 2099.7 | 9.27 |
| Laiwu | 238.67 | 1298.5 | 18.38 |
| Langfang | 1140.862 | 4358.8 | 26.17 |
| Lanzhou | 936.616 | 3616.2 | 25.90 |
| Lianyungang | 1070.188 | 4393.9 | 24.36 |
| Liaocheng | 695.739 | 5789.9 | 12.02 |
| Liaoyang | 620.288 | 1858.8 | 33.37 |
| Liaoyuan | 209.678 | 1176.6 | 17.82 |
| Lijiang | 128.848 | 1245 | 10.35 |
| Linfen | 635.71 | 4316.6 | 14.73 |
| Lishui | 448.88 | 2117 | 21.20 |
| Liu'an | 379.776 | 5612 | 6.77 |
| Liuzhou | 1106.096 | 3758.7 | 29.43 |
| Longnan | 131.365 | 2567.7 | 5.12 |
| Loudi | 501.615 | 3785.6 | 13.25 |
| Lvliang | 361.492 | 3727.1 | 9.70 |
| Ma'anshan | 516.606 | 1366 | 37.82 |
| Maoming | 620.851 | 5817.8 | 10.67 |
| Mianyang | 747.878 | 4613.9 | 16.21 |
| Mudanjiang | 23.65 | 2798.7 | 0.85 |
| Nanchang | 1235.277 | 5042.6 | 24.50 |
| Nanchong | 930.867 | 6278.6 | 14.83 |
| Nanjing | 4714.89 | 8004.7 | 58.90 |
| Nanning | 1930.216 | 6661.6 | 28.98 |
| Nantong | 2258.199 | 7282.8 | 31.01 |
| Ningbo | 7823.121 | 7605.7 | 102.86 |
| Panjin | 345.817 | 1392.5 | 24.83 |
| Pu'er | 233.122 | 2543 | 9.17 |
| Qingyang | 165.467 | 2211.2 | 7.48 |
| Qingyuan | 1631.227 | 3698.4 | 44.11 |
| Qinhuangdao | 525.617 | 2987.6 | 17.59 |
| Qinzhou | 242.058 | 3079.7 | 7.86 |
| Qiqihar | 614.791 | 5367 | 11.46 |
| Qujing | 518.546 | 5855 | 8.86 |
| Quzhou | 805.094 | 2122.7 | 37.93 |
| Sanya | 470.121 | 685.4 | 68.59 |
| Shangrao | 468.295 | 6579.7 | 7.12 |
| Shantou | 583.743 | 5391 | 10.83 |
| Shaoxing | 1871.129 | 4912.2 | 38.09 |
| Shaoyang | 521.997 | 7071.8 | 7.38 |
| Shenyang | 3397.203 | 8106.2 | 41.91 |
| Shiyan | 607.559 | 3340.8 | 18.19 |
| Shizuishan | 216.019 | 725.5 | 29.78 |
| Shuozhou | 226.59 | 1714.9 | 13.21 |
| Siping | 194.77 | 3386.3 | 5.75 |
| Suzhou | 7157.478 | 10466 | 68.39 |
| Suzhou | 333.895 | 5353 | 6.24 |
| Taian | 1085.073 | 5494.2 | 19.75 |
| Taiyuan | 2481.991 | 4201.6 | 59.07 |
| Taizhou | 1540.62 | 4618.6 | 33.36 |
| Taizhou | 2617.891 | 5968.8 | 43.86 |
| Tangshan | 1647.621 | 7577.3 | 21.74 |
| Tianjin | 5684.847 | 12938.2 | 43.94 |
| Tieling | 648.938 | 2717.7 | 23.88 |
| Tonghua | 333.637 | 2325.2 | 14.35 |
| Tongliao | 362.058 | 3139.2 | 11.53 |
| Tongling | 347.393 | 724 | 47.98 |
| Turpan | 115.743 | 622.7 | 18.59 |
| Ulanqab | 288.938 | 2143.6 | 13.48 |
| Weifang | 2003.457 | 9086.2 | 22.05 |
| Weihai | 1097.634 | 2804.8 | 39.13 |
| Wenzhou | 2416.055 | 9122.1 | 26.49 |
| Wuhai | 216.1 | 532.9 | 40.55 |
| Wuhan | 4789.449 | 9785.4 | 48.94 |
| Wuhu | 828.802 | 2263 | 36.62 |
| Wuwei | 153.369 | 1815.1 | 8.45 |
| Wuxi | 3983.419 | 6372.6 | 62.51 |
| Wuzhong | 169.305 | 1273.8 | 13.29 |
| Xiangtan | 497.425 | 2748.6 | 18.10 |
| Xiangyang | 842.67 | 5500.3 | 15.32 |
| Xiaogan | 467.471 | 4814.5 | 9.71 |
| Xilin Gol League | 261.29 | 1028 | 25.42 |
| Xingtai | 731.288 | 7104.1 | 10.29 |
| Xining | 395.352 | 2208.7 | 17.90 |
| Xinzhou | 417.393 | 3067.5 | 13.61 |
| Xuancheng | 438.373 | 2533 | 17.31 |
| Ya'an | 297.842 | 1507.2 | 19.76 |
| Yancheng | 1400.917 | 7260.2 | 19.30 |
| Yangquan | 280.312 | 1368.5 | 20.48 |
| Yangzhou | 1427.845 | 4459.8 | 32.02 |
| Yantai | 3138.85 | 6968.2 | 45.05 |
| Yibin | 830.897 | 4059.7 | 20.47 |
| Yichang | 1112.95 | 4059.7 | 27.41 |
| Yichun | 346.149 | 1148.1 | 30.15 |
| Yinchuan | 782.664 | 1993.1 | 39.27 |
| Yingkou | 875.818 | 2428.5 | 36.06 |
| Yingtan | 121.118 | 1124.9 | 10.77 |
| Yiyang | 384.624 | 4313.1 | 8.92 |
| Yongzhou | 380.187 | 5180.2 | 7.34 |
| Yueyang | 605.061 | 5477.9 | 11.05 |
| Yulin | 400.719 | 5487.4 | 7.30 |
| Yuncheng | 596.231 | 5134.8 | 11.61 |
| Yunfu | 266.894 | 2360.1 | 11.31 |
| Yuxi | 283.435 | 2304 | 12.30 |
| Zaozhuang | 508.935 | 3729.3 | 13.65 |
| Zhangjiajie | 131.962 | 1476.5 | 8.94 |
| Zhangjiakou | 846.879 | 4345.5 | 19.49 |
| Zhangye | 99.579 | 1199.5 | 8.30 |
| Zhaoqing | 827.695 | 3918.1 | 21.12 |
| Zhaotong | 236.61 | 5213 | 4.54 |
| Zhenjiang | 1012.385 | 3113.3 | 32.52 |
| Zhongshan | 3035.102 | 3120.9 | 97.25 |
| Zhongwei | 133.203 | 1080.8 | 12.32 |
| Zhoushan | 424.164 | 1121.3 | 37.83 |
| Zhuzhou | 665.169 | 3855.6 | 17.25 |
| Zibo | 1521.334 | 4530.6 | 33.58 |
| Zigong | 467.664 | 2678.9 | 17.46 |
| Ziyang | 218.918 | 3665.1 | 5.97 |

^*^ The number of city residents is according to the 2010 census.
