# Supplementary material for: Association between temperature variability and daily hospital admissions for cause-specific cardiovascular disease in urban China: A national time-series study
Source: PLoS Med. 2019 Jan 28;16(1):e1002738. doi: 10.1371/journal.pmed.1002738 (PMC6349307; doi:10.1371/journal.pmed.1002738)
Supplement: S2 Table — (DOCX) [file pmed.1002738.s003.docx]

**S2 Table:** Summary statistics on annual-average hospital admissions for cause-specific cardiovascular disease in 184 Chinese cities, 2014–2017.

| City | Cardiovascular disease | Ischemic heart disease | Heart failure | Heart rhythm disturbances | Ischemic stroke |
| --- | --- | --- | --- | --- | --- |
| An'shan | 72.87 | 43.76 | 2.28 | 1.93 | 31.25 |
| Anyang | 49.21 | 30.66 | 0.15 | 0.82 | 23.32 |
| Baicheng | 3.45 | 2.79 | 0.08 | 0.59 | 2.13 |
| Baise | 12.88 | 8.15 | 1.39 | 0.88 | 2.63 |
| Baiyin | 7.87 | 5.62 | 0.13 | 0.73 | 1.35 |
| Baoji | 109.73 | 75.20 | 4.28 | 6.07 | 31.69 |
| Baoshan | 7.15 | 3.75 | 0.25 | 0.63 | 3.25 |
| Baotou | 40.69 | 24.54 | 0.95 | 2.29 | 17.17 |
| Bayannur | 15.98 | 8.35 | 1.80 | 0.20 | 7.73 |
| Bazhong | 4.34 | 2.04 | 0.16 | 0.36 | 2.17 |
| Beihai | 3.13 | 2.06 | 0.12 | 0.32 | 0.54 |
| Bengbu | 12.92 | 8.40 | 0.35 | 1.63 | 3.72 |
| Benxi | 62.53 | 38.54 | 0.01 | 1.45 | 29.93 |
| Binzhou | 9.38 | 5.17 | 0.09 | 0.50 | 4.70 |
| Bozhou | 14.24 | 7.72 | 0.12 | 0.32 | 6.87 |
| Changde | 27.09 | 18.01 | 0.15 | 2.52 | 7.43 |
| Changsha | 120.38 | 89.36 | 0.24 | 2.78 | 32.54 |
| Changzhi | 29.63 | 11.54 | 0.27 | 1.04 | 19.93 |
| Changzhou | 33.97 | 13.14 | 0.92 | 1.68 | 24.24 |
| Chaoyang | 54.06 | 35.73 | 1.28 | 1.08 | 17.11 |
| Chengde | 28.64 | 16.02 | 0.36 | 1.31 | 6.81 |
| Chengdu | 97.85 | 70.30 | 1.83 | 6.51 | 17.48 |
| Chenzhou | 39.86 | 27.83 | 0.11 | 1.00 | 13.83 |
| Chifeng | 28.75 | 14.99 | 0.25 | 3.97 | 12.46 |
| Chizhou | 2.01 | 1.69 | 0.21 | 0.18 | 1.03 |
| Chongqing | 295.58 | 203.94 | 0.76 | 8.36 | 98.97 |
| Chongzuo | 2.56 | 1.89 | 0.04 | 0.22 | 0.47 |
| Chuzhou | 18.60 | 9.49 | 0.72 | 0.82 | 10.40 |
| Dalian | 198.97 | 125.09 | 4.35 | 7.77 | 77.25 |
| Dandong | 69.89 | 54.45 | 1.37 | 1.27 | 14.95 |
| Datong | 44.33 | 28.07 | 0.12 | 1.75 | 16.93 |
| Daxinganling | 9.38 | 8.06 | 0.29 | 0.22 | 1.11 |
| Dazhou | 11.41 | 4.77 | 0.11 | 2.21 | 6.22 |
| Dezhou | 33.58 | 20.75 | 0.21 | 0.86 | 15.62 |
| Dongying | 9.44 | 6.35 | 0.18 | 0.57 | 2.59 |
| Erdos | 11.41 | 5.80 | 0.10 | 0.58 | 6.48 |
| Fangchenggang | 3.31 | 1.89 | 0.05 | 0.17 | 1.08 |
| Fushun | 117.37 | 85.25 | 0.09 | 0.83 | 40.93 |
| Guangyuan | 12.02 | 6.76 | 0.14 | 2.95 | 2.95 |
| Guangzhou | 144.05 | 71.01 | 2.46 | 8.85 | 82.07 |
| Guigang | 3.37 | 2.03 | 0.12 | 0.11 | 1.20 |
| Guyuan | 2.52 | 1.88 | 0.05 | 0.11 | 0.49 |
| Haikou | 11.42 | 6.73 | 0.25 | 0.50 | 2.47 |
| Handan | 114.27 | 96.24 | 0.31 | 1.67 | 20.67 |
| Hangzhou | 74.66 | 41.58 | 1.45 | 6.97 | 32.88 |
| Hefei | 35.41 | 30.49 | 1.57 | 3.35 | 9.29 |
| Hegang | 12.80 | 5.68 | 0.32 | 0.32 | 8.22 |
| Heihe | 7.73 | 7.40 | 0.02 | 0.09 | 0.37 |
| Hengshui | 5.32 | 2.85 | 0.02 | 0.24 | 0.95 |
| Hengyang | 25.70 | 17.51 | 0.05 | 0.61 | 9.10 |
| Hetian | 7.43 | 6.04 | 0.02 | 0.60 | 0.23 |
| Heze | 36.70 | 23.44 | 0.26 | 0.99 | 15.97 |
| Hezhou | 8.22 | 4.79 | 0.66 | 0.93 | 2.01 |
| Hinggan League | 29.78 | 15.78 | 0.17 | 0.96 | 17.56 |
| Hohhot | 23.37 | 13.37 | 0.08 | 3.68 | 6.61 |
| Huai'an | 51.71 | 24.85 | 0.42 | 1.42 | 31.28 |
| Huaibei | 23.70 | 14.64 | 0.16 | 0.87 | 10.39 |
| Huaihua | 33.90 | 23.19 | 0.10 | 1.47 | 12.29 |
| Huainan | 33.45 | 27.26 | 0.53 | 0.55 | 2.67 |
| Huangshan | 5.77 | 2.81 | 0.55 | 0.91 | 2.05 |
| Huludao | 53.10 | 33.91 | 0.79 | 1.21 | 21.32 |
| Hulunbeier | 33.88 | 17.83 | 0.48 | 1.40 | 18.14 |
| Jiaxing | 21.66 | 10.38 | 0.91 | 3.48 | 9.15 |
| Jiayuguan | 6.56 | 4.92 | 0.02 | 0.28 | 0.84 |
| Jilin | 89.39 | 55.10 | 0.26 | 2.30 | 35.22 |
| Jinan | 98.07 | 64.75 | 1.58 | 3.40 | 37.76 |
| Jincheng | 23.20 | 12.05 | 0.10 | 0.37 | 13.93 |
| Jinhua | 21.68 | 15.61 | 0.35 | 1.70 | 5.31 |
| Jining | 93.32 | 61.85 | 0.38 | 2.39 | 38.15 |
| Jinzhong | 16.02 | 6.82 | 0.20 | 0.92 | 12.22 |
| Jinzhou | 64.61 | 39.09 | 1.01 | 1.65 | 30.64 |
| Jixi | 20.58 | 17.31 | 0.31 | 0.18 | 3.46 |
| Karamay | 1.24 | 1.17 | NA | 0.02 | 0.02 |
| Kashi | 16.42 | 14.63 | 0.16 | 0.39 | 0.22 |
| Kiamusze | 111.77 | 61.76 | 0.15 | 0.59 | 10.30 |
| Kunming | 32.11 | 32.11 | NA | NA | NA |
| Laibin | 2.97 | 2.03 | 0.08 | 0.15 | 0.68 |
| Laiwu | 23.11 | 17.34 | 0.04 | 0.32 | 7.11 |
| Langfang | 11.62 | 8.26 | 0.08 | 0.52 | 1.24 |
| Lanzhou | 27.09 | 18.35 | 0.49 | 2.88 | 4.48 |
| Lianyungang | 14.95 | 7.81 | 0.33 | 1.09 | 3.31 |
| Liaocheng | 49.19 | 30.06 | 0.02 | 1.27 | 24.70 |
| Liaoyang | 86.10 | 46.09 | 1.76 | 4.67 | 49.66 |
| Liaoyuan | 19.24 | 6.67 | 0.06 | 0.53 | 11.86 |
| Lijiang | 4.09 | 2.94 | 0.03 | 0.20 | 1.13 |
| Linfen | 18.17 | 16.98 | 0.24 | 0.95 | 7.29 |
| Lishui | 4.60 | 4.60 | NA | NA | NA |
| Liu'an | 16.84 | 6.59 | 0.37 | 0.47 | 8.74 |
| Liuzhou | 20.70 | 19.14 | 0.30 | 1.27 | 8.39 |
| Longnan | 1.33 | 1.28 | NA | NA | 0.02 |
| Loudi | 12.38 | 9.31 | 0.02 | 0.43 | 2.97 |
| Lvliang | 3.35 | 2.30 | 0.02 | 0.26 | 0.59 |
| Ma'anshan | 14.95 | 6.29 | 0.95 | 2.18 | 6.77 |
| Maoming | 4.51 | 3.84 | 0.03 | 0.04 | 0.18 |
| Mianyang | 3.32 | 2.57 | 0.12 | 0.16 | 0.05 |
| Mudanjiang | 3.23 | 2.15 | 0.05 | 0.16 | 0.51 |
| Nanchang | 31.31 | 16.06 | 0.68 | 2.89 | 14.07 |
| Nanchong | 12.01 | 6.10 | 0.08 | 1.58 | 3.52 |
| Nanjing | 146.68 | 57.31 | 4.26 | 6.76 | 104.37 |
| Nanning | 18.68 | 8.30 | 1.67 | 1.28 | 3.49 |
| Nantong | 78.63 | 37.91 | 1.23 | 5.23 | 45.59 |
| Ningbo | 23.06 | 11.74 | 0.91 | 3.84 | 8.71 |
| Panjin | 31.76 | 22.51 | 0.23 | 0.57 | 10.93 |
| Pu'er | 14.19 | 6.87 | 0.19 | 0.44 | 8.44 |
| Qingyang | 11.63 | 6.81 | 0.06 | 1.28 | 4.11 |
| Qingyuan | 27.67 | 14.20 | 2.62 | 1.62 | 10.49 |
| Qinhuangdao | 23.58 | 14.71 | 0.21 | 1.38 | 9.36 |
| Qinzhou | 13.29 | 5.86 | 0.85 | 1.09 | 5.42 |
| Qiqihar | 18.06 | 8.64 | NA | 0.05 | 3.86 |
| Qujing | 17.01 | 10.35 | 1.67 | 0.86 | 5.35 |
| Quzhou | 14.37 | 7.62 | 0.50 | 1.76 | 5.76 |
| Sanya | 8.85 | 3.46 | 0.01 | 0.23 | 2.79 |
| Shangrao | 12.35 | 5.61 | 0.21 | 0.69 | 7.27 |
| Shantou | 14.80 | 6.52 | 1.54 | 1.45 | 6.35 |
| Shaoxing | 14.29 | 6.98 | 0.42 | 2.13 | 6.14 |
| Shaoyang | 34.85 | 20.07 | 0.29 | 0.88 | 16.26 |
| Shenyang | 368.21 | 271.28 | 0.05 | 3.38 | 124.50 |
| Shiyan | 6.75 | 5.02 | 0.06 | 0.45 | 1.95 |
| Shizuishan | 11.78 | 8.16 | 0.32 | 0.28 | 2.50 |
| Shuozhou | 8.29 | 3.94 | 0.01 | 0.23 | 5.67 |
| Siping | 19.45 | 9.93 | 0.07 | 0.48 | 12.61 |
| Suzhou | 42.11 | 12.66 | 1.86 | 6.12 | 28.63 |
| Suzhou | 17.02 | 11.16 | 0.07 | 0.63 | 7.09 |
| Taian | 65.98 | 44.60 | 0.29 | 1.56 | 25.96 |
| Taiyuan | 66.31 | 45.29 | 1.68 | 1.54 | 2.39 |
| Taizhou | 32.88 | 15.30 | 0.47 | 2.69 | 4.26 |
| Taizhou | 5.57 | 2.67 | 0.26 | 0.89 | 2.10 |
| Tangshan | 53.29 | 36.31 | 0.65 | 2.85 | 15.70 |
| Tianjin | 302.25 | 168.43 | 13.22 | 16.00 | 124.65 |
| Tieling | 83.44 | 53.30 | 0.59 | 0.99 | 40.79 |
| Tonghua | 19.12 | 10.80 | 0.06 | 0.37 | 9.57 |
| Tongliao | 17.72 | 11.20 | 0.05 | 1.11 | 8.69 |
| Tongling | 5.04 | 2.77 | 0.30 | 0.34 | 1.37 |
| Turpan | 8.27 | 6.69 | 0.02 | 0.29 | 1.75 |
| Ulanqab | 13.71 | 7.17 | 0.10 | 0.55 | 8.36 |
| Weifang | 72.64 | 35.20 | 12.43 | 7.03 | 23.48 |
| Weihai | 22.18 | 16.51 | 0.34 | 0.99 | 5.71 |
| Wenzhou | 11.75 | 6.00 | 0.70 | 1.68 | 4.38 |
| Wuhai | 20.00 | 8.54 | 0.38 | 0.77 | 12.32 |
| Wuhan | 159.49 | 96.34 | 3.39 | 7.69 | 59.69 |
| Wuhu | 47.40 | 35.52 | 0.40 | 1.91 | 12.09 |
| Wuwei | 4.33 | 3.87 | 0.05 | 0.23 | 0.15 |
| Wuxi | 35.13 | 20.50 | 0.66 | 3.90 | 13.41 |
| Wuzhong | 6.94 | 5.19 | 0.03 | 0.51 | 1.27 |
| Xiangtan | 32.34 | 24.82 | 0.05 | 0.62 | 8.35 |
| Xiangyang | 13.59 | 12.38 | 0.05 | 0.41 | 1.10 |
| Xiaogan | 7.11 | 4.57 | 0.15 | 0.32 | 1.99 |
| Xilin Gol League | 6.33 | 5.09 | 0.05 | 0.26 | 1.47 |
| Xingtai | 21.15 | 16.42 | 0.69 | 1.23 | 2.97 |
| Xining | 7.50 | 4.66 | 1.05 | 1.80 | 2.39 |
| Xinzhou | 16.53 | 9.13 | 0.19 | 0.77 | 9.60 |
| Xuancheng | 11.99 | 6.53 | 0.30 | 1.66 | 4.38 |
| Ya'an | 4.36 | 2.59 | 0.10 | 0.62 | 0.82 |
| Yancheng | 51.97 | 23.13 | 1.99 | 1.55 | 32.39 |
| Yangquan | 14.16 | 7.78 | 0.07 | 0.38 | 6.69 |
| Yangzhou | 26.09 | 6.76 | 0.24 | 2.56 | 16.14 |
| Yantai | 37.01 | 11.19 | 1.89 | 4.46 | 25.27 |
| Yibin | 19.40 | 13.54 | 0.56 | 1.24 | 5.09 |
| Yichang | 26.62 | 17.45 | 0.49 | 2.52 | 8.16 |
| Yichun | 21.08 | 19.25 | 0.40 | 0.37 | 1.61 |
| Yinchuan | 14.40 | 8.89 | 0.26 | 0.57 | 3.19 |
| Yingkou | 82.13 | 55.77 | 0.97 | 5.93 | 25.81 |
| Yingtan | 3.41 | 1.63 | 0.06 | 0.10 | 0.56 |
| Yiyang | 27.20 | 14.32 | 2.76 | 0.26 | 8.55 |
| Yongzhou | 8.81 | 5.98 | 0.05 | 0.11 | 2.88 |
| Yueyang | 29.73 | 20.08 | 0.06 | 1.05 | 8.78 |
| Yulin | 12.12 | 6.91 | 0.31 | 0.56 | 4.41 |
| Yuncheng | 38.76 | 21.56 | 0.27 | 1.15 | 21.77 |
| Yunfu | 1.49 | 1.25 | 0.03 | 0.06 | 0.05 |
| Yuxi | 15.58 | 7.92 | 0.17 | 0.64 | 8.86 |
| Zaozhuang | 29.74 | 20.64 | 0.11 | 1.00 | 10.63 |
| Zhangjiajie | 8.28 | 5.56 | 0.01 | 0.31 | 2.98 |
| Zhangjiakou | 36.26 | 27.57 | 0.54 | 1.66 | 2.76 |
| Zhangye | 5.39 | 5.17 | NA | 0.08 | NA |
| Zhaoqing | 9.52 | 5.39 | 0.17 | 0.37 | 0.35 |
| Zhaotong | 7.14 | 4.24 | 0.08 | 0.28 | 3.33 |
| Zhenjiang | 7.43 | 4.57 | 0.20 | 0.64 | 2.65 |
| Zhongshan | 6.06 | 2.84 | 0.36 | 0.88 | 2.48 |
| Zhongwei | 3.52 | 2.51 | 0.04 | 0.34 | 0.44 |
| Zhoushan | 9.16 | 3.45 | 0.48 | 1.83 | 4.30 |
| Zhuzhou | 33.70 | 22.38 | 0.11 | 2.64 | 10.78 |
| Zibo | 110.94 | 90.95 | 1.17 | 1.26 | 23.37 |
| Zigong | 17.90 | 12.22 | 0.18 | 0.98 | 4.81 |
| Ziyang | 3.74 | 2.19 | 0.01 | 0.14 | 1.27 |

NA indicates data is not available in the city.
