# Supplementary material for: Association between temperature variability and daily hospital admissions for cause-specific cardiovascular disease in urban China: A national time-series study
Source: PLoS Med. 2019 Jan 28;16(1):e1002738. doi: 10.1371/journal.pmed.1002738 (PMC6349307; doi:10.1371/journal.pmed.1002738)
Supplement: S3 Table — TV0–1, temperature variability at 0–1 days. (DOCX) [file pmed.1002738.s004.docx]

**S3 Table:** Summary statistics on annual-average temperature variability at 0–1 days (TV_0–1_), weather conditions and air pollutants in 184 Chinese cities, 2014–2017.

| City | TV_0–1_  (°C) | Relative humidity (%) | Temperature  (°C) | PM_2.5_  (μg/m^3^) | SO_2_  (μg/m^3^) | NO_2_  (μg/m^3^) |
| --- | --- | --- | --- | --- | --- | --- |
| An'shan | 5.5 | 50 | 11.3 | 63.9 | 46.4 | 35.5 |
| Anyang | 5.7 | 59 | 15.1 | 91 | 53.7 | 51.5 |
| Baicheng | 7.2 | 56 | 5.5 | 59 | 19.9 | 19.3 |
| Baise | 4.9 | 78 | 23.2 | 40.3 | 16.1 | 17 |
| Baiyin | 8 | 55 | 11.8 | 37.3 | 43.2 | 27.5 |
| Baoji | 6.2 | 66 | 12.8 | 61.4 | 15.9 | 37.6 |
| Baoshan | 6.2 | 66 | 17.4 | 27.8 | 11.6 | 14.4 |
| Baotou | 8 | 55 | 8.4 | 48.5 | 37 | 42 |
| Bayannur | 7.5 | 45 | 7.3 | 40.9 | 28.6 | 27.7 |
| Bazhong | 4.6 | 75 | 17.5 | 35.1 | 4.9 | 28.2 |
| Beihai | 3.7 | 83 | 24.4 | 26.1 | 9.4 | 12.8 |
| Bengbu | 5.5 | 77 | 16.2 | 61 | 21.7 | 37.5 |
| Benxi | 6.7 | 60 | 9.1 | 48.9 | 37.1 | 36.1 |
| Binzhou | 5.7 | 61 | 14.3 | 77.1 | 47.8 | 41.9 |
| Bozhou | 5.5 | 71 | 16.6 | 60.4 | 27.6 | 35.2 |
| Changde | 4.5 | 78 | 18.1 | 55.7 | 21.1 | 21.6 |
| Changsha | 4.3 | 78 | 17.9 | 61.6 | 18.1 | 38.8 |
| Changzhi | 6.1 | 58 | 11.3 | 64.3 | 47.1 | 38.8 |
| Changzhou | 4.7 | 73 | 17.1 | 56.5 | 26.1 | 43.8 |
| Chaoyang | 8.1 | 50 | 10.1 | 41.6 | 32.4 | 22.1 |
| Chengde | 7.9 | 56 | 10.5 | 37.8 | 17.1 | 34.8 |
| Chengdu | 4.8 | 82 | 16.1 | 63.5 | 14.7 | 52.1 |
| Chenzhou | 4.4 | 79 | 18.6 | 41.3 | 16.9 | 25.9 |
| Chifeng | 7.5 | 48 | 8.4 | 39.3 | 38.9 | 21.9 |
| Chizhou | 4.7 | 77 | 17.5 | 53.7 | 18.5 | 31.3 |
| Chongqing | 3.8 | 77 | 18.6 | 56.4 | 16.6 | 43.5 |
| Chongzuo | 4.5 | 78 | 22.9 | 32.8 | 11.4 | 18 |
| Chuzhou | 5.2 | 80 | 16.5 | 57.6 | 15.4 | 37.8 |
| Dalian | 4.1 | 61 | 12.7 | 43.2 | 24 | 32.6 |
| Dandong | 5.7 | 69 | 10.9 | 39 | 24.3 | 24.7 |
| Datong | 9 | 56 | 8.1 | 38.3 | 42.5 | 28.8 |
| Daxinganling | 8.1 | 67 | 1.9 | 20.6 | 26.3 | 17.6 |
| Dazhou | 4.4 | 77 | 18.3 | 55.2 | 11.5 | 39.6 |
| Dezhou | 6.4 | 67 | 13.8 | 89.1 | 39.4 | 42 |
| Dongying | 5.6 | 61 | 14.8 | 68.1 | 48.9 | 40.6 |
| Erdos | 5.9 | 47 | 8.2 | 25.7 | 17.6 | 24.9 |
| Fangchenggang | 4 | 81 | 23.6 | 28.4 | 9.5 | 16.6 |
| Fushun | 8.1 | 66 | 7.4 | 50.3 | 29.9 | 34.6 |
| Guangyuan | 5.3 | 69 | 16.4 | 24.4 | 20.3 | 35.1 |
| Guangzhou | 4.8 | 80 | 22 | 39.4 | 13.2 | 47.5 |
| Guigang | 4.3 | 82 | 22.7 | 39.8 | 17.1 | 23.8 |
| Guyuan | 7 | 55 | 7.8 | 36.5 | 12.7 | 26.5 |
| Haikou | 3.6 | 82 | 24.4 | 22.8 | 6.4 | 13.4 |
| Handan | 5.7 | 58 | 14.8 | 92.1 | 44.8 | 50.6 |
| Hangzhou | 4.7 | 74 | 17.9 | 52.6 | 14.6 | 45.8 |
| Hefei | 5.3 | 75 | 20.5 | 49.3 | 11.5 | 48.6 |
| Hegang | 7 | 66 | 6.1 | 36.7 | 9.8 | 15.4 |
| Heihe | 6.9 | 63 | 3.2 | 24.9 | 19.7 | 15.8 |
| Hengshui | 7.1 | 57 | 10.9 | 101 | 43.8 | 43.8 |
| Hengyang | 4.1 | 75 | 19.4 | 51.4 | 16.6 | 28.9 |
| Hetian | 6.3 | 36 | 14.5 | 118.4 | 39.5 | 27.8 |
| Heze | 6 | 69 | 14.9 | 86.1 | 37.6 | 39.5 |
| Hezhou | 4.9 | 78 | 21.5 | 36.6 | 14.6 | 16.3 |
| Hinggan League | 6.8 | 48 | 6.8 | 29.4 | 10.4 | 17.9 |
| Hohhot | 7.2 | 47 | 7.2 | 43.1 | 32.5 | 42.2 |
| Huai'an | 5.4 | 77 | 15.8 | 55 | 19.5 | 25.7 |
| Huaibei | 5.4 | 67 | 16.3 | 59.3 | 24.2 | 37.7 |
| Huaihua | 4.5 | 80 | 17.6 | 43.7 | 18.2 | 17.7 |
| Huainan | 5.5 | 77 | 16.5 | 56.9 | 17.9 | 31 |
| Huangshan | 3.7 | 80 | 9.5 | 27.9 | 13 | 18.5 |
| Huludao | 6.2 | 53 | 11.4 | 50.6 | 47.1 | 36.8 |
| Hulunbeier | 7 | 62 | -0.2 | 28 | 7 | 19.3 |
| Jiaxing | 4.1 | 82 | 17 | 48.6 | 18 | 40.4 |
| Jiayuguan | 7.8 | 47 | 8.6 | 27.6 | 20.4 | 26.6 |
| Jilin | 7.7 | 65 | 5.4 | 54.1 | 24.8 | 33.1 |
| Jinan | 5.5 | 56 | 15.4 | 79.2 | 44.1 | 50.3 |
| Jincheng | 6.1 | 59 | 10.8 | 62.1 | 57.6 | 40.4 |
| Jinhua | 4.8 | 74 | 18.8 | 51.6 | 19.8 | 37.6 |
| Jining | 6.5 | 70 | 14.6 | 73.9 | 49.6 | 43.6 |
| Jinzhong | 7.6 | 59 | 11.7 | 60.2 | 81.4 | 37.3 |
| Jinzhou | 6.2 | 53 | 11.1 | 55.7 | 53.2 | 38.4 |
| Jixi | 6.1 | 64 | 6.8 | 33.1 | 15.1 | 20.2 |
| Karamay | 5.6 | 47 | 11.1 | 29.5 | 6.8 | 22.1 |
| Kashi | 6.5 | 46 | 11.5 | 155.3 | 14.7 | 34.1 |
| Kiamusze | 6.4 | 71 | 7.8 | 25.9 | 12.1 | 23.2 |
| Kunming | 6.2 | 70 | 16 | 29.4 | 17.2 | 30.4 |
| Laibin | 4.6 | 79 | 22.2 | 42.9 | 20 | 20.5 |
| Laiwu | 6 | 62 | 13.7 | 81.4 | 56.9 | 48.3 |
| Langfang | 7.4 | 61 | 11 | 71.9 | 18.8 | 54.2 |
| Lanzhou | 9.1 | 58 | 10.2 | 51.5 | 19.3 | 53.2 |
| Lianyungang | 5.2 | 73 | 16.7 | 43.2 | 19 | 32.1 |
| Liaocheng | 6.1 | 65 | 13.6 | 88.9 | 35.6 | 42.7 |
| Liaoyang | 7.5 | 61 | 9.2 | 51.3 | 28.2 | 30.8 |
| Liaoyuan | 7.7 | 66 | 7.3 | 48 | 22.9 | 28.5 |
| Lijiang | 6.8 | 59 | 13.8 | 14.8 | 9.4 | 12.9 |
| Linfen | 6.4 | 54 | 14.5 | 88.6 | 93.3 | 38.6 |
| Lishui | 5.6 | 74 | 19.2 | 36.1 | 10.9 | 26.8 |
| Liu'an | 4.9 | 76 | 16.8 | 50.2 | 12.9 | 30.6 |
| Liuzhou | 4.3 | 72 | 22.1 | 45.3 | 19.1 | 25.3 |
| Longnan | 6.6 | 67 | 14.5 | 25.9 | 19 | 22.3 |
| Loudi | 4.8 | 80 | 18.2 | 46.6 | 23.4 | 22.9 |
| Lvliang | 7.7 | 60 | 11.2 | 61.8 | 76 | 43 |
| Ma'anshan | 4.8 | 77 | 16.9 | 56.1 | 20.6 | 33.7 |
| Maoming | 4 | 84 | 19.4 | 37.9 | 12.6 | 16.6 |
| Mianyang | 4.2 | 72 | 17.8 | 68.3 | 18.1 | 43.5 |
| Mudanjiang | 7 | 65 | 5.5 | 35.5 | 14.1 | 25.3 |
| Nanchang | 4.1 | 75 | 19.3 | 43.4 | 20.4 | 32 |
| Nanchong | 4.1 | 79 | 18 | 61.1 | 16.7 | 32.1 |
| Nanjing | 4.8 | 73 | 16.7 | 54.6 | 19.1 | 48 |
| Nanning | 4.7 | 81 | 20.7 | 37.4 | 12.9 | 34.3 |
| Nantong | 4.8 | 78 | 16.4 | 50.9 | 25.6 | 36.5 |
| Ningbo | 4.7 | 76 | 17.9 | 41.3 | 15.1 | 40.3 |
| Panjin | 7 | 60 | 9.8 | 46.5 | 26.3 | 27.9 |
| Pu'er | 6 | 76 | 19.6 | 25.6 | 8.2 | 15.5 |
| Qingyang | 5.5 | 59 | 10.5 | 35.7 | 32.3 | 20.8 |
| Qingyuan | 4.4 | 77 | 22.1 | 37.6 | 18.3 | 35.7 |
| Qinhuangdao | 6.1 | 68 | 11.2 | 48.9 | 35.7 | 47.7 |
| Qinzhou | 4 | 81 | 23.5 | 34.3 | 17.3 | 19.3 |
| Qiqihar | 6.2 | 62 | 9.4 | 33.1 | 19.5 | 22.5 |
| Qujing | 6.1 | 70 | 15.6 | 31 | 22.6 | 21 |
| Quzhou | 5 | 79 | 18.5 | 45.4 | 18.9 | 33.1 |
| Sanya | 2.9 | 92 | 22.8 | 17.7 | 2.3 | 13.3 |
| Shangrao | 5.3 | 78 | 18.6 | 42.6 | 38.8 | 30.3 |
| Shantou | 3.8 | 77 | 23.2 | 32.6 | 13.6 | 21.1 |
| Shaoxing | 4.7 | 73 | 17.9 | 51.8 | 22.8 | 41.9 |
| Shaoyang | 4.5 | 81 | 17.5 | 55.6 | 31.6 | 22.5 |
| Shenyang | 7.5 | 60 | 9.1 | 61.2 | 54.6 | 44.3 |
| Shiyan | 6 | 70 | 16.1 | 49.9 | 20.2 | 29.3 |
| Shizuishan | 7.6 | 46 | 11.2 | 45.5 | 61.6 | 30.3 |
| Shuozhou | 8.7 | 52 | 8.8 | 52.6 | 59.6 | 33.4 |
| Siping | 6.9 | 63 | 7.9 | 51.6 | 24.4 | 33 |
| Suzhou | 4.8 | 78 | 16.4 | 53.1 | 18.3 | 50.7 |
| Suzhou | 5.4 | 72 | 16.6 | 65.9 | 20.7 | 36.6 |
| Taian | 3.9 | 65 | 6.7 | 67.3 | 38.1 | 40.8 |
| Taiyuan | 8.3 | 48 | 5.6 | 70.8 | 68.9 | 33.9 |
| Taizhou | 4.9 | 78 | 17 | 69 | 30.4 | 23.4 |
| Taizhou | 4.3 | 78 | 18.7 | 39 | 8.9 | 24.1 |
| Tangshan | 7.2 | 63 | 11.8 | 83.7 | 54.3 | 60.3 |
| Tianjin | 6.2 | 57 | 13.8 | 71.3 | 29.3 | 47.9 |
| Tieling | 7.4 | 61 | 8.3 | 51.6 | 23.2 | 31.3 |
| Tonghua | 7.1 | 67 | 6.7 | 42.8 | 32 | 32.8 |
| Tongliao | 6.9 | 52 | 8.5 | 42.7 | 15.3 | 22.4 |
| Tongling | 4.4 | 81 | 18.3 | 53.3 | 35.1 | 43.1 |
| Turpan | 6.8 | 34 | 16.5 | 67.3 | 16.1 | 38.8 |
| Ulanqab | 7.6 | 47 | 5.6 | 35.7 | 26.1 | 29.7 |
| Weifang | 6.1 | 62 | 14.4 | 68.5 | 40.4 | 36.4 |
| Weihai | 4.1 | 62 | 13.9 | 35.3 | 15.9 | 21.8 |
| Wenzhou | 3.2 | 81 | 18.2 | 41.6 | 14.1 | 44.1 |
| Wuhai | 7.6 | 44 | 10.9 | 48.2 | 56 | 28.5 |
| Wuhan | 5.3 | 80 | 16.1 | 66.8 | 18.8 | 50.9 |
| Wuhu | 4.8 | 78 | 17.2 | 56.2 | 20.3 | 39.6 |
| Wuwei | 7.4 | 44 | 10.4 | 41.3 | 18.3 | 28.7 |
| Wuxi | 4.8 | 74 | 17.2 | 56.4 | 21.3 | 44.5 |
| Wuzhong | 7.4 | 47 | 10.8 | 47.2 | 33.1 | 24 |
| Xiangtan | 4.6 | 81 | 17.3 | 59.2 | 25.1 | 37.5 |
| Xiangyang | 5.1 | 71 | 16.9 | 67.4 | 16.2 | 33.8 |
| Xiaogan | 5 | 76 | 18.3 | 46.1 | 10.4 | 24.5 |
| Xilin Gol League | 8.3 | 53 | 4.7 | 15.7 | 18.1 | 13.5 |
| Xingtai | 5.7 | 59 | 16.6 | 87.2 | 49.6 | 56.6 |
| Xining | 8.9 | 58 | 6.7 | 43.9 | 28.7 | 42 |
| Xuancheng | 4.8 | 79 | 17.7 | 48.9 | 20.4 | 35 |
| Ya'an | 4.3 | 80 | 16.2 | 40.4 | 12.9 | 25.3 |
| Yancheng | 4.9 | 79 | 14.9 | 48.3 | 17 | 25.7 |
| Yangquan | 5.8 | 57 | 15.2 | 62.7 | 60.2 | 44.4 |
| Yangzhou | 4.5 | 73 | 16.5 | 56.7 | 27 | 31.2 |
| Yantai | 5.9 | 64 | 13.3 | 42.6 | 22.4 | 35.3 |
| Yibin | 4.1 | 77 | 19.1 | 58 | 22 | 30.3 |
| Yichang | 4.6 | 77 | 16.6 | 70.6 | 23.4 | 35.1 |
| Yichun | 7.4 | 68 | 2 | 22.7 | 9 | 15.4 |
| Yinchuan | 7.3 | 49 | 9.5 | 51 | 59.4 | 38.7 |
| Yingkou | 5.2 | 63 | 10.6 | 44 | 25.2 | 31.2 |
| Yingtan | 4.4 | 82 | 15.2 | 44.1 | 35.2 | 26.8 |
| Yiyang | 3.9 | 78 | 17.3 | 45.8 | 23 | 29.2 |
| Yizhou | 6.9 | 48 | 10.8 | 59.3 | 51.4 | 39 |
| Yongzhou | 4.2 | 81 | 18.7 | 47.6 | 21.2 | 25 |
| Yueyang | 3.5 | 79 | 17.9 | 52.4 | 22.8 | 25.5 |
| Yulin | 4.4 | 81 | 22.4 | 37.7 | 25.3 | 23.6 |
| Yuncheng | 6.8 | 60 | 14.9 | 67.2 | 57.2 | 33.8 |
| Yunfu | 4.4 | 83 | 24.1 | 36.3 | 15 | 31.6 |
| Yuxi | 6.4 | 71 | 16.9 | 25.7 | 18.6 | 20.4 |
| Zaozhuang | 5.8 | 64 | 15 | 78.9 | 47 | 33.3 |
| Zhangjiajie | 5 | 81 | 17.1 | 48.5 | 9.8 | 19.1 |
| Zhangjiakou | 7.4 | 50 | 11.7 | 32.7 | 17.2 | 25.7 |
| Zhangye | 9.3 | 41 | 9.5 | 35 | 17.8 | 24.9 |
| Zhaoqing | 3.8 | 71 | 16.4 | 80 | 36.2 | 46.4 |
| Zhaotong | 6.3 | 75 | 12.7 | 31.9 | 25.3 | 18.3 |
| Zhenjiang | 4.5 | 72 | 16.5 | 58.3 | 22.4 | 42.4 |
| Zhongshan | 4 | 78 | 23 | 34.2 | 13.1 | 32.6 |
| Zhongwei | 8.3 | 50 | 10.4 | 42.9 | 24.8 | 21.8 |
| Zhoushan | 4.2 | 81 | 17.5 | 27.5 | 8.5 | 19.3 |
| Zhuzhou | 4.5 | 79 | 18.3 | 57.7 | 24.5 | 35.1 |
| Zibo | 6 | 62 | 13.8 | 80.5 | 74.6 | 56.6 |
| Zigong | 4.3 | 82 | 17.4 | 72.8 | 18.2 | 31.1 |
| Ziyang | 4.5 | 79 | 17.4 | 43.7 | 21 | 21.5 |
