# Supplementary material for: Association between temperature variability and daily hospital admissions for cause-specific cardiovascular disease in urban China: A national time-series study
Source: PLoS Med. 2019 Jan 28;16(1):e1002738. doi: 10.1371/journal.pmed.1002738 (PMC6349307; doi:10.1371/journal.pmed.1002738)
Supplement: S4 Table — (DOCX) [file pmed.1002738.s005.docx]

**S4 Table.** The distribution of daily temperature variability (TV) at different exposure days in 184 cities in China, 2014–2017.

|  |  |  | Percentile | | |  |
| --- | --- | --- | --- | --- | --- | --- |
| Variable | Mean ± SD | Minimum | 25th | 50th | 75th | Maximum |
| TV_0–1_ (°C) | 5.7 ± 2.2 | 0.5 | 4.1 | 5.6 | 7.2 | 15.5 |
| TV_0–2_ (°C) | 5.6 ± 2.0 | 0.6 | 4.2 | 5.5 | 6.9 | 14.7 |
| TV_0–3_ (°C) | 5.6 ± 1.9 | 0.7 | 4.2 | 5.5 | 6.8 | 13.9 |
| TV_0–4_ (°C) | 5.6 ± 1.8 | 0.7 | 4.3 | 5.5 | 6.8 | 13.5 |
| TV_0–5_ (°C) | 5.6 ± 1.7 | 0.7 | 4.3 | 5.5 | 6.8 | 12.7 |
| TV_0–6_ (°C) | 5.6 ± 1.7 | 0.7 | 4.4 | 5.5 | 6.8 | 12.4 |
| TV_0–7_ (°C) | 5.6 ± 1.6 | 0.9 | 4.4 | 5.5 | 6.8 | 12.2 |
