# Supplementary material for: Association between temperature variability and daily hospital admissions for cause-specific cardiovascular disease in urban China: A national time-series study
Source: PLoS Med. 2019 Jan 28;16(1):e1002738. doi: 10.1371/journal.pmed.1002738 (PMC6349307; doi:10.1371/journal.pmed.1002738)
Supplement: S5 Table — CI, confidence interval; PC, percentage change; TV0–1, temperature variability at 0–1 days. (DOCX) [file pmed.1002738.s006.docx]

**S5 Table.** National-average percentage change with 95% confidence interval in daily hospital admissions for cardiovascular disease associated with 1 °C increase in temperature variability at 0–1 days in 184 Chinese cities by climate region, 2014–2017.

| Areas | Percentage change | 95% confidence interval | *P* |
| --- | --- | --- | --- |
| Cold area (≤ 10.6 °C) | 0.38 | 0.15-0.62 | 0.001 |
| Moderate cold area (10.6–15.5 °C) | 0.60 | 0.34-0.86 | <0.001 |
| Moderate hot area (15.5–17.9 °C) | 0.43 | 0.13-0.73 | 0.005 |
| Hot area (> 17.9 °C) | 0.55 | 0.18-0.93 | 0.004 |
