# Supplementary material for: Association between temperature variability and daily hospital admissions for cause-specific cardiovascular disease in urban China: A national time-series study
Source: PLoS Med. 2019 Jan 28;16(1):e1002738. doi: 10.1371/journal.pmed.1002738 (PMC6349307; doi:10.1371/journal.pmed.1002738)
Supplement: S6 Table — CI, confidence interval; PC, percentage change; TV0–1, temperature variability at 0–1 days. (DOCX) [file pmed.1002738.s007.docx]

**S6 Table.** National-average percentage change with 95% confidence interval in daily hospital admissions for cause-specific cardiovascular disease per 1 °C increase in temperature variability at 0–1 days if temperature variability > 3 °C in 184 Chinese cities, 2014–2017.

| Areas | Percentage change | 95% confidence interval | *P* |
| --- | --- | --- | --- |
| Cardiovascular disease | 0.48 | 0.33-0.64 | < 0.001 |
| Ischemic heart disease | 0.33 | 0.20-0.46 | <0.001 |
| Heart failure | 0.51 | 0.04-0.99 | 0.032 |
| Heart rhythm disturbances | 0.35 | 0.03-0.67 | 0.031 |
| Ischemic stroke | 0.89 | 0.58-1.20 | < 0.001 |
