# Supplementary material for: Association between temperature variability and daily hospital admissions for cause-specific cardiovascular disease in urban China: A national time-series study
Source: PLoS Med. 2019 Jan 28;16(1):e1002738. doi: 10.1371/journal.pmed.1002738 (PMC6349307; doi:10.1371/journal.pmed.1002738)
Supplement: S7 Table — CI, confidence interval; IQR, interquartile range; PC, percentage change; TV0–1, temperature variability at 0–1 days. (DOCX) [file pmed.1002738.s008.docx]

**S7 Table.** National-average percentage change with 95% confidence interval in daily hospital admissions for cause-specific cardiovascular disease per interquartile range increase in temperature variability at 0–1 days (3.1 °C) in 184 Chinese cities, 2014–2017.

| Areas | Percentage change | 95% confidence interval | *P* |
| --- | --- | --- | --- |
| Cardiovascular disease | 1.42 | 0.92-1.93 | <0.001 |
| Ischemic heart disease | 0.93 | 0.55-1.30 | <0.001 |
| Heart failure | 1.45 | 0.03-2.87 | 0.044 |
| Heart rhythm disturbances | 1.06 | 0.05-2.07 | 0.040 |
| Ischemic stroke | 2.24 | 1.53-2.96 | <0.001 |
