# Supplementary material for: Association between temperature variability and daily hospital admissions for cause-specific cardiovascular disease in urban China: A national time-series study
Source: PLoS Med. 2019 Jan 28;16(1):e1002738. doi: 10.1371/journal.pmed.1002738 (PMC6349307; doi:10.1371/journal.pmed.1002738)
Supplement: S8 Table — CI, confidence interval; PC, percentage change; TV0–1, temperature variability at 0–1 days. (DOCX) [file pmed.1002738.s009.docx]

**S8 Table.** National-average percentage change with 95% confidence interval in daily hospital admissions for cardiovascular disease associated with 1 °C increase in temperature variability at 0–1 days in 184 Chinese cities, 2014–2017, classified by city-specific annual average NO_2_ levels.

| Areas | Percentage change | 95% confidence interval | *P* |
| --- | --- | --- | --- |
| Low-polluted area (≤ 25.8 μg/m^3^) | 0.19 | -0.10-0.48 | 0.200 |
| Moderate-polluted area (25.8–36.5 μg/m^3^) | 0.58 | 0.32-0.84 | <0.001 |
| High-polluted area (> 36.5 μg/m^3^) | 0.57 | 0.33-0.81 | <0.001 |
