# Supplementary figures and images for: Association between temperature variability and daily hospital admissions for cause-specific cardiovascular disease in urban China: A national time-series study
Source: PLoS Med. 2019 Jan 28;16(1):e1002738. doi: 10.1371/journal.pmed.1002738 (PMC6349307; doi:10.1371/journal.pmed.1002738)

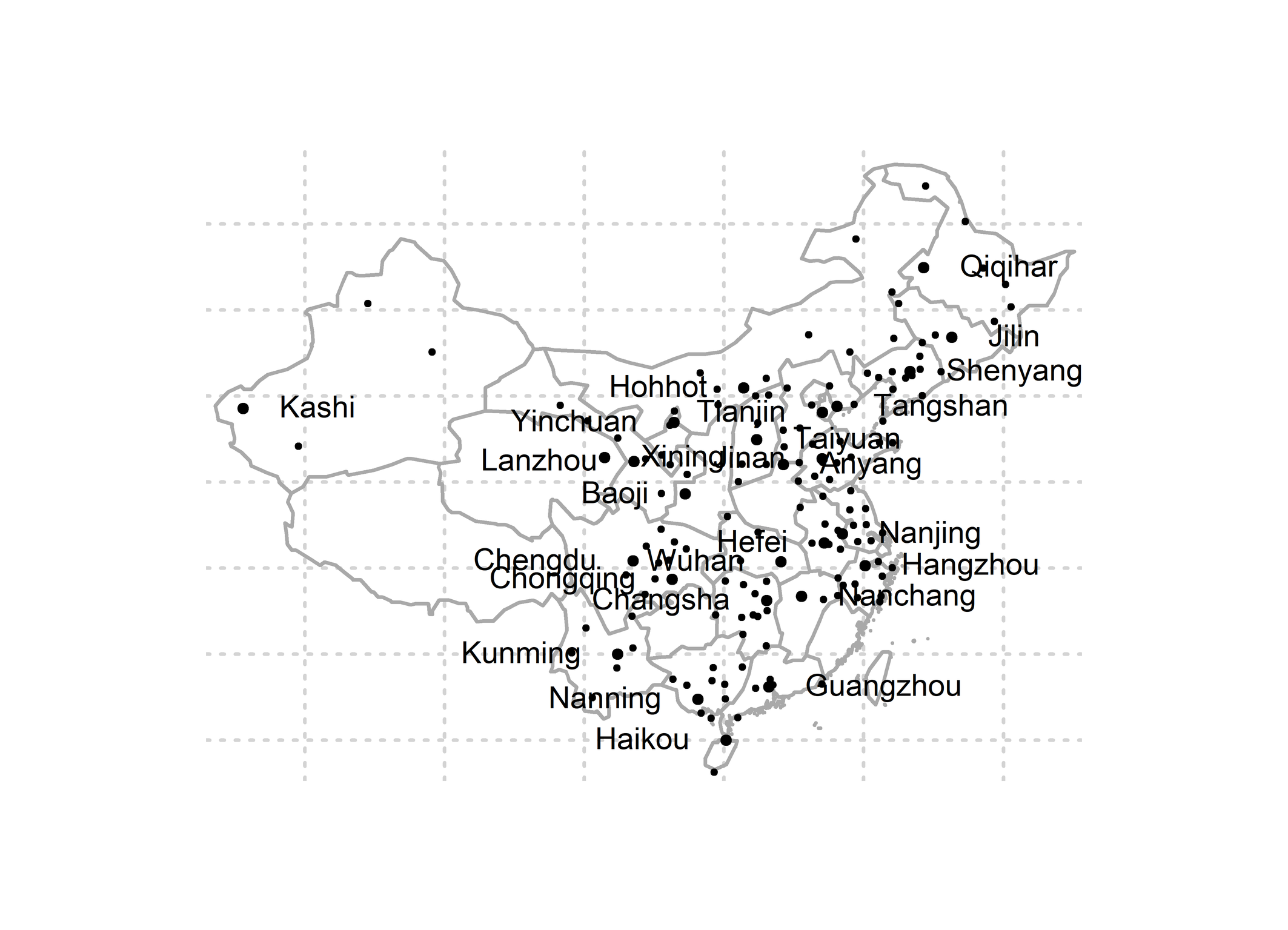

Supplement: S1 Fig — The most populous city in each province was marked by name. (TIF) [file pmed.1002738.s010.tif]
