# Supplementary material for: Association between temperature variability and daily hospital admissions for cause-specific cardiovascular disease in urban China: A national time-series study
Source: PLoS Med. 2019 Jan 28;16(1):e1002738. doi: 10.1371/journal.pmed.1002738 (PMC6349307; doi:10.1371/journal.pmed.1002738)
Supplement: S1 Appendix — (DOCX) [file pmed.1002738.s011.docx]

**Statistical Analyses Plan**

1. **Objective**

To assess the association between the temperature variability and daily hospital admissions for cause-specific cardiovascular disease.

1. **Exposure**

The temperature variability (TV) is defined as the standard deviation (SD) of daily minimum and maximum temperatures over the exposure days. The TV variables include TV_0-1_, TV_0-2_, TV_0-3_, TV_0-4_, TV_0-5_, TV_0-6_ and TV_0-7_.

1. **Outcomes**

The outcomes to be investigated include hospital admissions of cardiovascular disease, ischemic heart disease, heart failure, heart rhythm disturbances, and ischemic stroke.

1. **Method**

The associations between TV and daily hospital admissions for the outcome is estimated by a two-stage approach. In the first stage, generalized linear models (GLMs) with quasi-Poisson regression is going to be applied for estimating city-specific estimates of hospital admissions associated with TV exposure. In the second stage, the pooled estimates of the associations between TV and hospital admissions are calculated by combining city-specific estimates using a random-effects meta-analysis.

1. **Primary analysis**

In the first stage, linear term for TV will be included in the model according to the previous studies. Confounding risk factors are considered as follows:

- Long-term time trends: a natural cubic spline of calendar time with 7 degrees of freedom (*df*) per year;
- Relative humidity: a natural cubic spline of 3-day moving average relative humidity with 3 *df*;
- Day of the week and public holiday: indicator variables;
- Daily mean temperature: distributed lag non-linear model (DLNM). Specifically, natural smooth functions of daily mean temperature and lags over time up to 21 days with 4 *df* to accommodate the non-linear and lagged effects of ambient temperature. Three internal knots are placed at equally spaced temperature percentiles (25th, 50th, and 75th).

Consequently, the model is as shown below:

Log[E(Y_t_)] = α + *β* (temperature variability) + γ Temperature + day of the week + public holidays + *ns*(calendar time, *df* = 7/per year) + *ns*(relative humidity, *df* = 3)

Where *β* represents the log-relative risk of admission associated with a unit increase of temperature variability; E(Y_t_) is the expected count of admissions on day t; Temperature indicates a two dimensional cross-basis matrix produced by DLNM; *ns*() indicates natural cubic spline function; public holidays and day of the week were included in the model as indicator variables; relative humidity indicates 3-day moving average relative humidity.

**Primary statistics to report**: percentage change and 95% CI in hospital admissions per 1-°C increase in temperature variability. Percentage change equals relative risk minus 1 and then multiplies by 100.

**Shape of association**

A natural cubic spline of TV will be used to investigate the shape association between TV_0-1_ and hospital admissions. Relative change and 95% CIs are plotted against TV_0-1_.

**Between-study heterogeneity**

Between-study heterogeneity will be assessed by the *I*^2^ statistic, which quantifies the percentage of variance in the point estimates of the study-specific effect estimates that is attributable to between-study variation as opposed to sampling variation (with values close to 0 indicating lack of evidence of heterogeneity).

**Potential Effect modification**

For cities’ characteristics, city-specific relative risk (and their CIs) as the outcome are meta-regressed on each continuous variable of city characteristics. City-level characteristics include annual-average temperature variability levels, temperature and relative humidity, gross domestic product (GDP) per capita, and coverage of population.

Effect modification by individual characteristics are investigated by stratified analyses. The subgroup variables include sex, age groups (18–64, 65–74 and ≥75 years) and geographical region (north and south regions).

1. **Sensitivity analysis**

The following sensitivity analyses will be conducted:

- Changing the maximum lag of temperature from 21 to 28 days
- Changing the *df* s for time (6–8 per year)
- Changing the *df* s for temperature (3–6)
- Adjusting for air pollutants (daily PM_2.5_, NO_2_ and SO_2_ concentrations) in the GLMs.

1. **Proposed Main Tables & Figures**

**Figure 1 Locations of the cities included in the study.**

**Table 1 Summary information of the city-level characteristics**

- City-specific number of people enrolled
- City-specific number of residents
- City-specific coverage rate based on the UEBMI

**Table 2 Summary statistics of health and environmental data during the study period.**

- Average and range of annual mean hospital admissions for CVD across the cities
- Average and range of annual mean relative humidity across the cities
- Average and range of annual mean temperature across the cities
- Average and range of annual mean TV at different exposure days from TV_0–1_ to TV_0–7_ across the cities

**Table 3 Primary analysis for the association between TV and CVD, and cause-specific cardiovascular disease.**

- Outcomes: CVD, ischemic heart disease, heart failure, heart rhythm disturbances and ischemic stroke
- Exposures: TV_0–1_ to TV_0–7_.

**Figure 2 Shape of association between TV_0-1_ and CVD**

**Table 4 Stratified analyses for the association between TV_0-1_ and cardiovascular disease**

- Subgroup variables: sex, age groups (18–64, 65–74 and ≥75 years) and geographical region (north and south regions).

**Table 5 Potential effect modifications of the association between TV_0-1_ and cardiovascular disease by city-level characteristics**

- City-level characteristics: annual-average temperature variability levels, temperature and relative humidity, GDP per capita, and coverage of population.

**Table 6 Results from the sensitivity analysis**

- Changing the maximum lag of temperature from 21 to 28 days
- Changing the *df*s for time (6–8 per year)
- Changing the *df*s for temperature (3–6)
- Adjusting for air pollutants (daily PM_2.5_, NO_2_ and SO_2_ concentrations) in the GLMs.
